# Assessing COVID-19 risk, vulnerability and infection prevalence in communities

**DOI:** 10.1371/journal.pone.0241166

**Published:** 2020-10-29

**Authors:** Amin Kiaghadi, Hanadi S. Rifai, Winston Liaw

**Affiliations:** 1 Civil and Environmental Engineering, University of Houston, Houston, TX, United States of America; 2 Oden Institute for Computational Engineering and Sciences, University of Texas Austin, Austin, TX, United States of America; 3 Health Systems and Population Health Sciences, College of Medicine, University of Houston, Houston, TX, United States of America; Columbia University, UNITED STATES

## Abstract

**Background:**

The spread of coronavirus in the United States with nearly five and half million confirmed cases and over 170,000 deaths has strained public health and health care systems. While many have focused on clinical outcomes, less attention has been paid to vulnerability and risk of infection. In this study, we developed a planning tool that examines factors that affect vulnerability to COVID-19.

**Methods:**

Across 46 variables, we defined five broad categories: 1) access to medical services, 2) underlying health conditions, 3) environmental exposures, 4) vulnerability to natural disasters, and 5) sociodemographic, behavioral, and lifestyle factors. The developed tool was validated by comparing the estimated overall vulnerability with the real-time reported normalized confirmed cases of COVID-19.

**Analysis:**

A principal component analysis was undertaken to reduce the dimensions. In order to identify vulnerable census tracts, we conducted rank-based exceedance and K-means cluster analyses.

**Results:**

All of the 5 vulnerability categories, as well as the overall vulnerability, showed significant (P-values <<0.05) and relatively strong correlations (0.203<ρ<0.57) with the normalized confirmed cases of COVID-19 at the census tract level. Our study showed a total of 722,357 (~17% of the County population) people, including 171,403 between the ages of 45–65 (~4% of County’s population), and 76,719 seniors (~2% of County population), are at a higher risk based on the aforementioned categories. The exceedance and K-means cluster analysis demonstrated that census tracts in the northeastern, eastern, southeastern and northwestern regions of the County are at highest risk.

**Conclusion:**

Policymakers can use this planning tool to identify neighborhoods at high risk for becoming hot spots; efficiently match community resources with needs, and ensure that the most vulnerable have access to equipment, personnel, and medical interventions.

## Introduction

The outbreak of the novel Coronavirus was first reported in Wuhan, China but has since spread to almost every country in the world. The highest number of cases and deaths, as of this writing, has been reported in the U. S. [1]. Within the 50 states, there is an apparent disparity in the number and causes of infections and their spread within each state. However, what is common to all cases reported thus far, is the rates of mortality and hospitalizations that appear to be highest among relatively older populations and populations with underlying medical conditions that facilitate morbidity due to COVID-19 [2–8].

Much research has focused on clinical outcomes, epidemiological modeling, and transmission dynamics of the novel coronavirus (see for example, [9–12]), but less focus has been placed on risk and vulnerability to contracting the disease. Emerging studies have begun to report on the impacts of social vulnerability on COVID-19 from an incidence and outcome standpoint [2–7, 13]. However, the spatial resolution of most studies to date has been at the global or country level, and less attention has been paid to finer spatial resolutions such as the census tract scale within a county. A finer spatial resolution is important from a vulnerability and risk standpoint as demonstrated in a recent study that showed that the poorest neighborhoods in Houston, Texas, might be at a higher risk of hospitalization from COVID-19 [14] based on an analysis of the Centers for Disease Control (CDC) underlying risk factors for severe COVID-19 cases [4] that include: asthma, Chronic Obstructive Pulmonary Disease (COPD), heart disease, hypertension, diabetes, and a history of heart attacks or strokes.

While the aforementioned underlying medical conditions are important risk factors, they weigh in on the risk of hospitalization but not necessarily on the risk of contracting the disease. As such, underlying medical conditions and sociodemographic variables may not fully represent the magnitude of the risk and the challenge in managing and mitigating disease in affected populations from pandemics such as COVID-19. Environmental pollutants such as air quality [15], CO_2_ emissions [13], and ambient conditions such as temperature and humidity [5, 16] showed correlations with COVID-19 morbidity. Furthermore, environmental exposures due to proximity to contaminated areas such as Superfund sites, hazardous waste sites, landfills, and leaky petroleum tanks has long-term adverse effects on public health, immune systems, and vulnerability to certain diseases [17–21]. Public health is further exacerbated by natural disasters, such as hurricanes and severe storms [22–24] that expose populations to pathogens and pollutants in floodwater and their flooded homes and potentially contribute to weakened immune systems. Behavioral and lifestyle factors could also affect the vulnerability of a population to an infectious disease such as COVID-19. Obesity, in recent COVID-19 data, has been shown to be prevalent in hospitalized patients [7], and smoking has been associated with disease progression [25]. Finally, it should be noted that because the risk is unevenly distributed, shortages in hospital beds, personal protective equipment (PPE), and medications have emerged in some but not all communities [26–28], thereby widening disparities and exposing systemic shortcomings [29]. Limited access to medical services, especially with less than fully-functional transportation systems combined with lack of insurance coverage, could worsen the impact of COVID-19 for people with less favorable sociodemographic metrics and people in rural regions. Thus, a more holistic view of the vulnerability of communities to COVID-19 that considers all of the aforementioned variables is needed to guide decision-makers in identifying the areas and populations in their jurisdictions that require specific resources, response, and mitigation actions.

While there are numerous indices with different applications, none, by themselves, can provide a comprehensive description of the influential factors in the COVID-19 pandemic. Among the existing indices, the Social Vulnerability Index (SVI) developed by the Geospatial Research, Analysis, and Services Program (GRASP) at the Centers for Disease Control and Prevention (CDC) and Agency for Toxic Substances and Disease Registry [30] is the most commonly used one and has been used to explain the variability in COVID-19 spread [6]. While SVI considers socioeconomic status, household composition/disability, minority status/language, and housing/transportation, it does not take environmental exposure, underlying medical conditions, behavioral, and lifestyle factors, and vulnerability to natural hazards into account. Moreover, access to medical services, which is a key factor in a pandemic, is not included in SVI. The same comments are valid for other similar indices such as Concentrated Disadvantage Measures [31], and the Social Deprivation Index [32]; these indices have not been developed with a pandemic such as COVID-19 in mind, but rather for identifying disparities in population with regards to specific determinants. In this study, we develop a rigorous planning tool at the census tract level that examines influential determinants of vulnerability to COVID-19 in 5 broad categories (with 46 variables) that include: 1) access to medical services, 2) underlying medical conditions, 3) environmental exposures, 4) vulnerability to natural disasters and 5) sociodemographic, behavioral, and lifestyle factors. To the best of the knowledge of the authors, none of the existing studies provide a holistic perspective on COVID-19 vulnerability. The goals for developing the planning tool are to better understand medical access gaps and identify parts of the county where more protective measures and response actions need to be put in place.

Such a planning tool is critical in order to mitigate the impact of COVID-19 and prepare for future pandemics. Using this tool, policymakers can identify neighborhoods with a higher potential for becoming the next hot spots, efficiently match community resources with community needs, and ensure that equipment, personnel, medications, and support are available to everyone, particularly the most vulnerable and those in greatest need. This strategy is essential to address historical trends that have preferentially delivered resources to those with means resulting in gaps in quality [33–35]. The planning framework developed in the study is readily transferable to other counties in the US and can be expanded to the state level for decision-making on a short-term or long-term basis towards improving the overall health of communities in each state.

## Methods

### Study region

**Fig 1:**
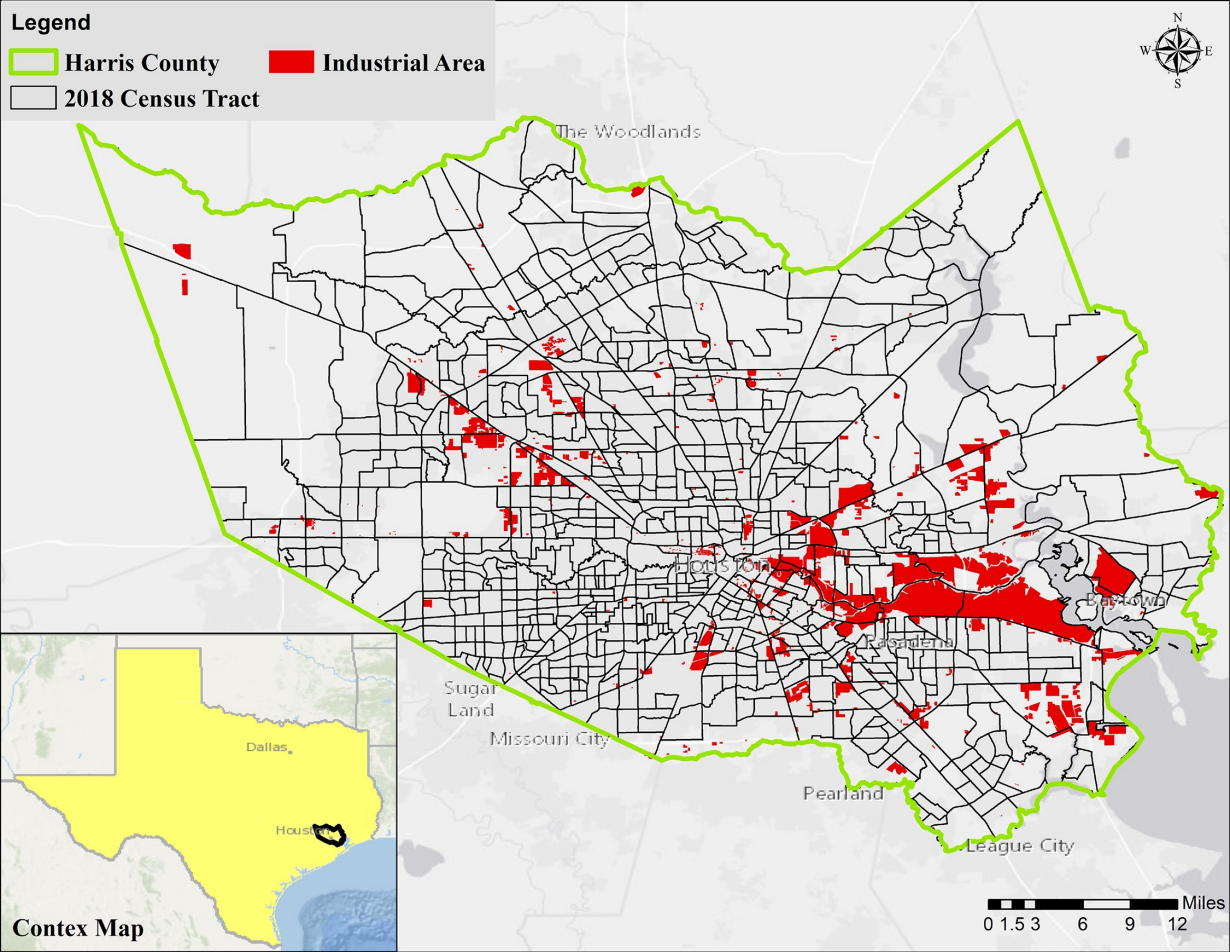
Map of Harris County in Texas and its 786 census tracts (2018). The industrial areas are defined according to the State of Texas classification of parcels.

**Fig 2:**
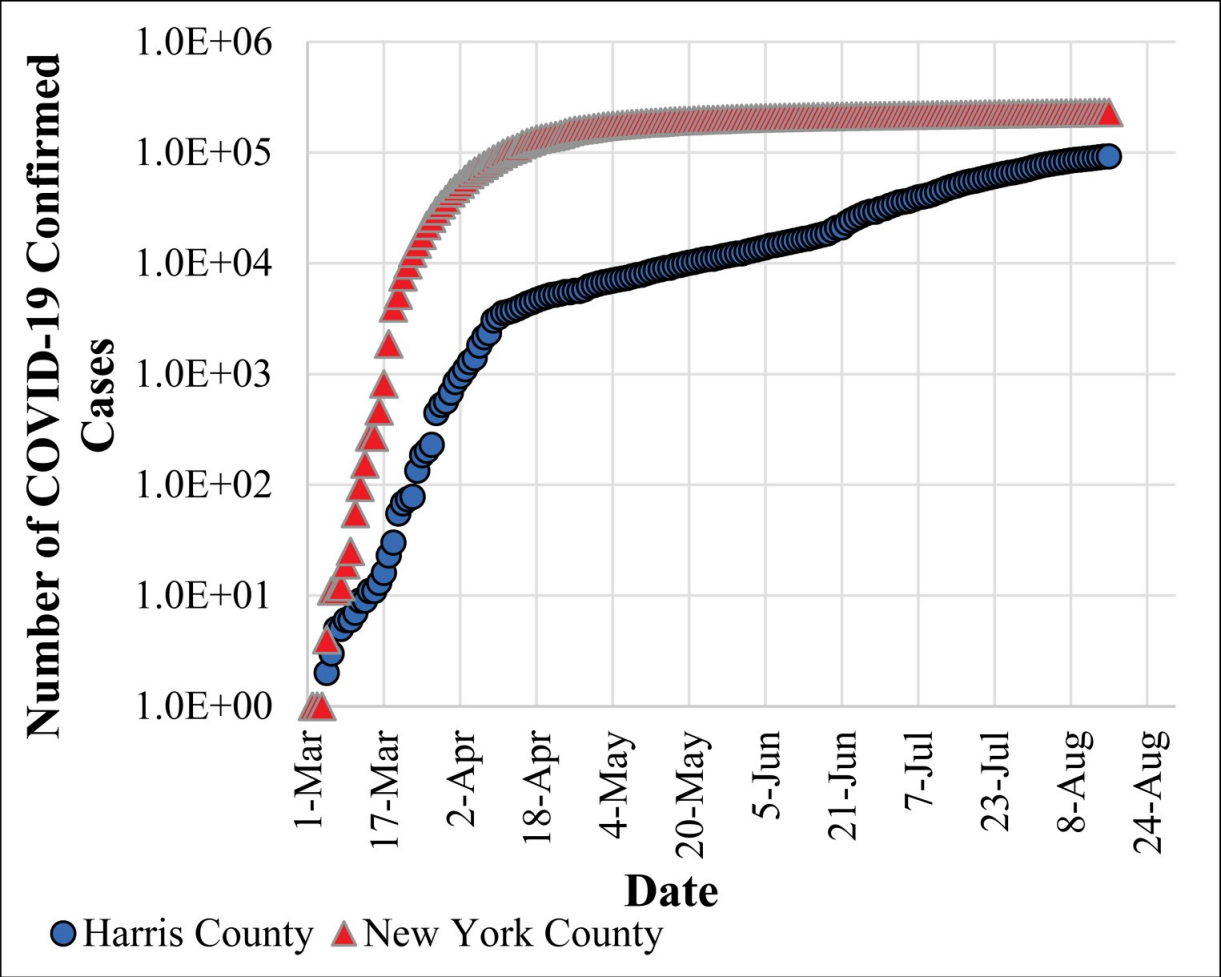
Number of confirmed cases of COVID-19 over time since March 1, 2020 in New York County and Harris County.

Harris County, located in the southeastern part of Texas (Fig 1), is the third-most populous county in the U.S., with more than 4.7 million residents [36]. While ranked number 2 in the nation in terms of Gross Domestic Product (GDP) growth, the County exhibits geospatial socioeconomic disparities among its population. While Harris County was experiencing fewer cases, and lower rates of transmission relative to the rest of the U.S., starting around mid-June 2020, the pandemic resurged, and the number of COVID-19 cases has increased substantially. Fig 2 shows the number of confirmed cases of COVID-19 in Harris County compared to New York County, for example. As shown in Fig 2, both the total number of confirmed cases [1] and the slope of the spread during the initial phase of the spread are significantly higher in New York compared to Harris County. However, over time, the spread curve was flattened in New York while a second surge wave of the pandemic occurred in Harris County. This is important to note because it potentially offers the County the opportunity for using the developed tool for improved long-term planning to respond to community health needs and disparities in response to COVID-19 and other pandemics or natural disasters.

### Data acquisition and processing

The total number of confirmed COVID-19 cases at the county level was downloaded from the Johns Hopkins Center for Civic Impact’s Coronavirus resource center [1]. For Harris County and Nueces County, the total number of confirmed COVID-19 cases at the zip code levels were compiled from the Harris County Public Health database [37] and the City of Corpus Christi COVID-19 dashboard [38]. The zip code-level cases were converted to tract-level using the “Spatial Join” tool in ArcMap and then were normalized by dividing the number of cases to the population of each tract. All census data (2018) at the census tract level were compiled from the National Historical Geographic Information System (NHGIS) database [39]. Total population, the number of households, median income and income per capita (adjusted to 2018 U.S. dollars), percent of the population below the poverty line, with cash public assistance or food stamps/SNAP (Supplemental Nutrition Assistance Program), living alone, with health insurance coverage, with a disability, education, and age distribution for each tract in Harris County were accessed. Using the detailed variables in the census data, education in this study was defined as the percent of the population with high school diplomas or higher degrees. Due to the importance of age in the vulnerability to COVID-19, both median age and the percent of the population in decadal age intervals were calculated. The percent of the population below the poverty line was chosen as the main economic variable, and the household density was calculated by dividing the total number of households by the area of each census tract.

Two measures of vulnerability to flooding were defined, using data from Hurricane Harvey that had severe impacts on Harris County in 2017: i) the ratio of the number of households that filed damage claims based on Federal Emergency Management Agency (FEMA) data [40] to the total number of houses in each tract, and ii) the ratio of the wetted areas (with water depth greater than zero) during Hurricane Harvey in a census tract to the total area of the tract (the specific methodology for this approach is described in [41]).

Locations and types of medical facilities including nursing facilities, federally qualified health centers, hospitals, rural health clinics, urgent care centers, and Harris County Health System facilities were obtained from the Health Resources and Services Administration (HRSA) query data explorer tool [42], Harris County Health System [43], and the Homeland Infrastructure Foundation-Level Data (HIFLD) database [44]. The Microsoft Bing Maps Platform APIs [45] was used to estimate the drive time from the centroid of each tract to all of the available medical facilities nearby. ArcMap was used to extract the coordinates of both origins (centroids) and destinations (medical facilities), and the minimum travel time in minutes was then recorded for each tract in Microsoft Excel.

The underlying conditions that might affect the vulnerability to COVID-19 (arthritis, asthma, high blood pressure (HBP), cancer (except skin cancer), high cholesterol, chronic kidney and heart diseases, COPD, diabetes, poor physical and mental health, and stroke); as well as increased-risk behaviors/conditions (binge drinking, smoking, no leisure time physical activity, obesity, sleep less than 7 hours), and preventive indicators (annual doctor and dentist checkups, medication for high blood pressure (HBP), cholesterol screening, and routine physical exams) were all acquired from the 500 cities mapper database [46] (Table 1), which draws from the Centers for Disease Control and Prevention’s (CDC) Behavioral Risk Factor Surveillance System. It should be noted that data from [46] were only available at 584 out of 786 (73.8%) census tracts in Harris County. Census tracts without data are clearly identified in all figures.

**Table 1:** Variables within each category (the choice of variables was based on the PCA analysis, previous studies, and data availability).

Category	Name	Variables
1	Access to Medical Services	Household density, drive time to a medical facility, access to HBP medications^1^, physical checkup^1^, dental checkup^1^, cholesterol screening^1^, insurance coverage^1^, routine physical exams ^1^^,^^2^
2	Underlying Medical Conditions	Arthritis, Asthma, HBP, Cancer (except skin cancer), high cholesterol, chronic kidney disease, COPD, chronic heart disease, diabetes, poor mental condition, poor physical condition, stroke, at least one disability, median age, age above 50, age above 60, age above 70, age above 80
3	Environmental Exposures	Distance to a hazardous site, number of hazardous pollution events and LPST^3^, number of dry cleaners, petroleum storage tanks, and IHWCA sites^4^, ozone concentration, NO_2_ concentration, PM_2.5_ concentration
4	Vulnerability to Natural Disasters	FEMA Harvey claims ratio, Harvey inundation ratio
5	Sociodemographic, Behavioral, and Lifestyle Factors	Binge drinking, current smoker, no physical activity, obesity^5^, low sleep quality, education beyond high school diploma^1^, below the poverty line, living alone,

^1^ The exceedance was calculated in the opposite direction.

^2^ Routine physical exams include: Mammography (ages 50–74), Pap Smear use (ages 21–65), Fecal Occult blood test, Sigmoidoscopy, or Colonoscopy (ages 50–75), older men and women (+65) up to date on core clinical preventive services.

^3^ Leaky petroleum storage tank (underground tank).

^4^ Industrial and Hazardous Waste Corrective Action defined by the Texas Commission on Environmental Quality.

^5^ Obesity has emerged as a critical factor in hospitalization from COVID-19. In the context of this analysis, it was separated from other medical conditions in Category 2.

As noted before, ambient conditions such as temperature and humidity could affect the spread of COVID-19; however, in this study, an ambient gradient in Harris County was neglected as the spatial change over the County is expected to be minimal. Three indicators of air quality: ozone, nitrogen dioxide (NO_2_), and particulate matter smaller than 2.5 micrometers (PM_2.5_), were downloaded from the Texas Air Monitoring Information System (TAMIS) database [47]. For ozone, the 8-hour average concentrations were calculated using IBM SPSS (version 26) for all of the available monitoring stations (40) and compared with the 70 ppb standard established by the United States Environmental Protection Agency (EPA). The number of exceedances of the EPA standard for each monitoring station over the period of 2000–2019 was then calculated. “Interpolation” tools in ArcMap were used to convert the median measured concentration for each station to a continuous raster to overcome the spatial sparsity in measurements. The generated raster was then used to calculate the concentration of ozone for each census tract using the “Zonal Statistics” tool in ArcMap. One particular station (695: UH Moody Tower) was removed from the ozone calculation due to its extremely low temporal resolution compared to the other stations. Similar approaches were taken for NO_2_ (hourly measurements for 21 stations with 100 ppm standard) and PM_2.5_ (averaged daily data for 12 stations with a 35 μg/m^3^ standard).

Environmental releases from various sources to air, water, soils in Harris County were obtained from the United States Coast Guard National Response Center database [48]. The total number of emissions, pollution spills, or contaminant discharge events that occurred during the period between 2000 and 2020 for each zip code was extracted from the database by combining different years, filtering the actual events for Harris County, and removing redundant data points. The “Spatial Join” tool in ArcMap was used to convert the total number of events in each zip code to a summed total number of events for each census tract. The resulting value was added to the number of leaking petroleum storage tanks (underground and aboveground tanks) reported by the Texas Commission on Environmental Quality (TCEQ) [49] in the tract. A hazardous sites shapefile was developed by merging two databases: the EPA Superfund Enterprise Management System database [50], and the Texas Commission on Environmental Quality (TCEQ) GIS database [49]. From the latter source, the locations of municipal solid waste sites/landfills were acquired. The “Near” tool in Arcmap was used to calculate the distance between the centroid of each census tract to the nearest aforementioned hazardous sites. A second environmental variable was defined as the sum of the total number of dry cleaners, petroleum storage tanks (all underground and aboveground tanks), and sites that are part of an Industrial and Hazardous Waste Corrective Action (IHWCA) program located within each census tract; data for those was obtained from [49]. Both Shapiro-Wilk and Kolmogorov-Smirnov tests conducted in IBM SPSS showed that none of the datasets were normally distributed.

### Defining categories

A Principal Component Analysis (PCA) with orthogonal rotation (Varimax with Kaiser Normalization) was conducted in IBM SPSS as the first step to reduce the dimensions. Due to the limitation in data availability, as noted before, the PCA was performed for data from 584 tracts with all available data. Eigenvalues from random values were generated and compared with the values in this study using a parallel analysis engine [51]. This comparison was made to determine the number of components that should be retained in the analysis; components with eigenvalues greater than the randomized method were kept. The first five components that could explain ~ 80% of the variability in the 46 variables showed eigenvalues larger than the ones generated by the engine. S1 Table in S1 File shows the most dominant variables in each component (category).

The choice of variables for the study (Table 1) was based on the results of the PCA in addition to findings reported in previous studies, and data availability. Category 1 includes access to medical services, including medical facilities, medications, and insurance coverage, routine checkups, and physical exams, as well as household density as a surrogate for interaction among individuals within each tract (e.g., how crowded grocery stores could be in the tract). Category 2 includes chronic diseases, medical conditions, disability that could potentially affect the vulnerability to COVID-19, and age distribution. For environmental exposure, pollution events from various sources, the 3-air quality indicators, and the presence of hazardous sites were included. Flooding from Hurricane Harvey was the only metric in Category 4, although this could be expanded in future work to include heat, drought, wildfires, and other natural disasters. Finally and for Category 5, a combination of social, economic, behavioral, and lifestyle factors that could potentially threaten the health of individuals during the COVID-19 pandemic was considered.

### Statistical analyses

#### Vulnerability analysis

**Fig 3:**
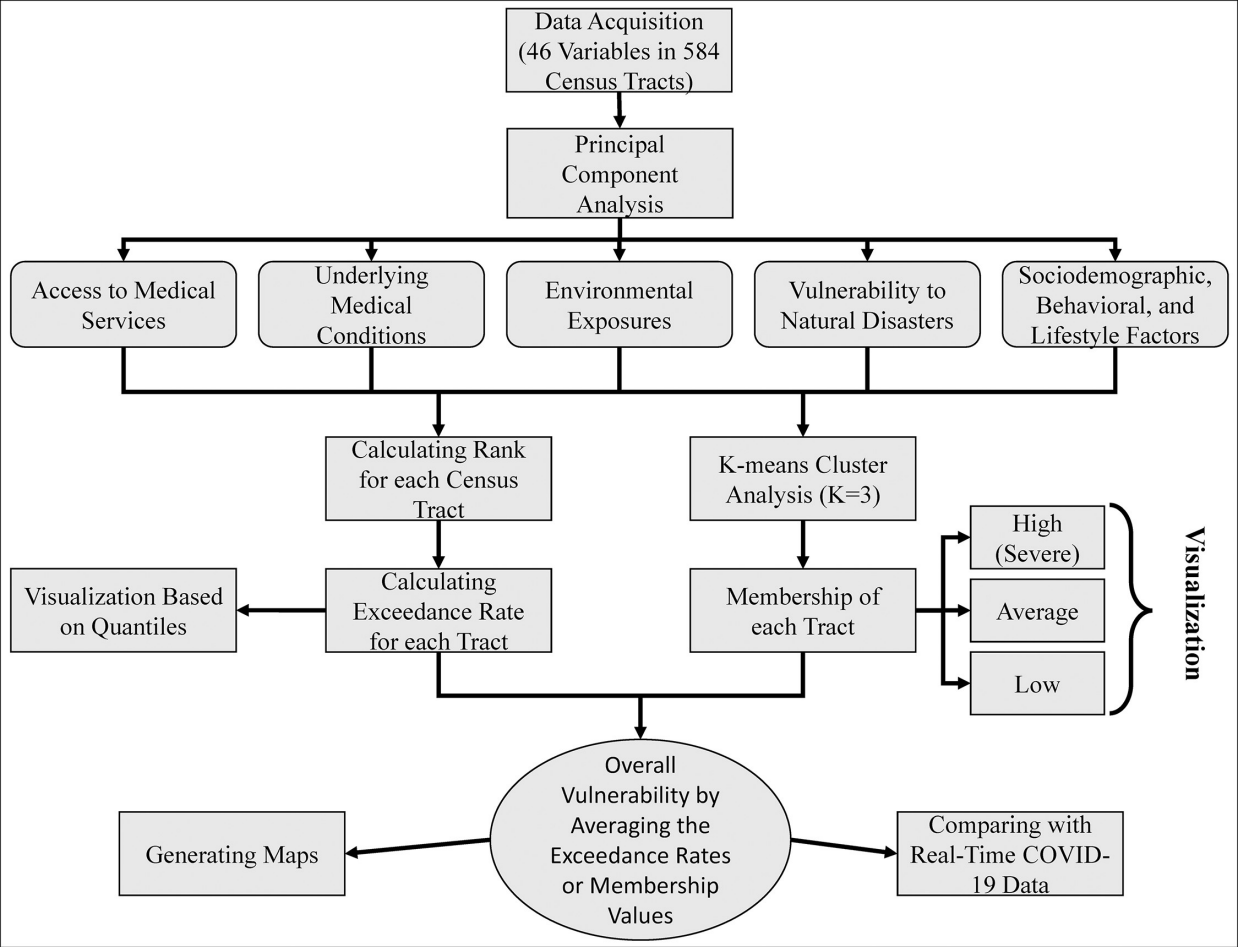
Flowchart showing the various steps in defining the five categories and calculating the vulnerabilities.

The step-by-step methodology applied in this study is depicted in Fig 3. Two classification approaches were used in the study with the goal of identifying the most vulnerable populations to COVID-19; a rank-based exceedance method developed in Microsoft Excel, and a standard K-Means Cluster Analysis (K = 3) using IBM SPSS. Validation of any developed models for the vulnerability was not possible due to lack of data at the desired spatial resolution and the fact that the pandemic is still developing. Thus, the second model (K-means) was used as a benchmark for the first model for comparison purposes.

In the rank-based exceedance method, for each variable, sorting the data in Microsoft Excel developed the rank of each census tract relative to other tracts within Harris County. The exceedance rate (percentile) was calculated as follows:

$$Exceedance=1-\frac{m}{n-1}$$ (1)

Where m is the rank, and n is the total number of tracts (786 in Harris County). The calculated exceedance for a given tract represents the percent of tracts that have a better condition than the selected one. To ensure that the direction of exceedance is the same among all variables, (1 –exceedance) was used for variables with positive nature such as insurance coverage, education, access to medication, and preventive tests. For each category, the average value of exceedance for all of the variables within that category was calculated and reported. In addition to classifying the tracts for each of the aforementioned categories, an overall vulnerability was defined by averaging the exceedance rates of the five defined categories. The percentile associated with each averaged value (for each category and for the overall vulnerability) was calculated and exported to ArcMap to generate decision-support level maps.

In the K-means cluster analysis (K-means is an unsupervised machine learning algorithm), three classes were defined for each category. As a result, the output classes were ordered as high (severe), average, and low depending on the order of the final cluster centers. The ANOVA test was conducted on the clusters to ensure that the values of the different variables were significantly different between clusters. Similar to the exceedance method, an overall vulnerability for each census tract was determined by averaging the five output class numbers (i.e., 1, 2, and 3) associated with the five categories. For illustration purposes, the percentile rank for each of the tracts was calculated and exported to ArcMap.

Although it is possible to assign weights to the categories and calculate a weighted average, equal importance for the categories was assumed in this study. Assigning weights is beyond the scope of this paper as there is not enough evidence to support such assignments as of this writing. Spearsman’s Correlation Analysis was performed to find correlations, if any, among the exceedance rates of vulnerabilities and normalized number of COVID-19 cases at the census tract level. Box plots were used to demonstrate the distribution of COVID-19 cases among the defined quantiles of Overall vulnerability using both K-means cluster analysis and exceedance methods.

## Results and discussion

### Geospatial distribution of determinants in the 5 categories

**Fig 4:**
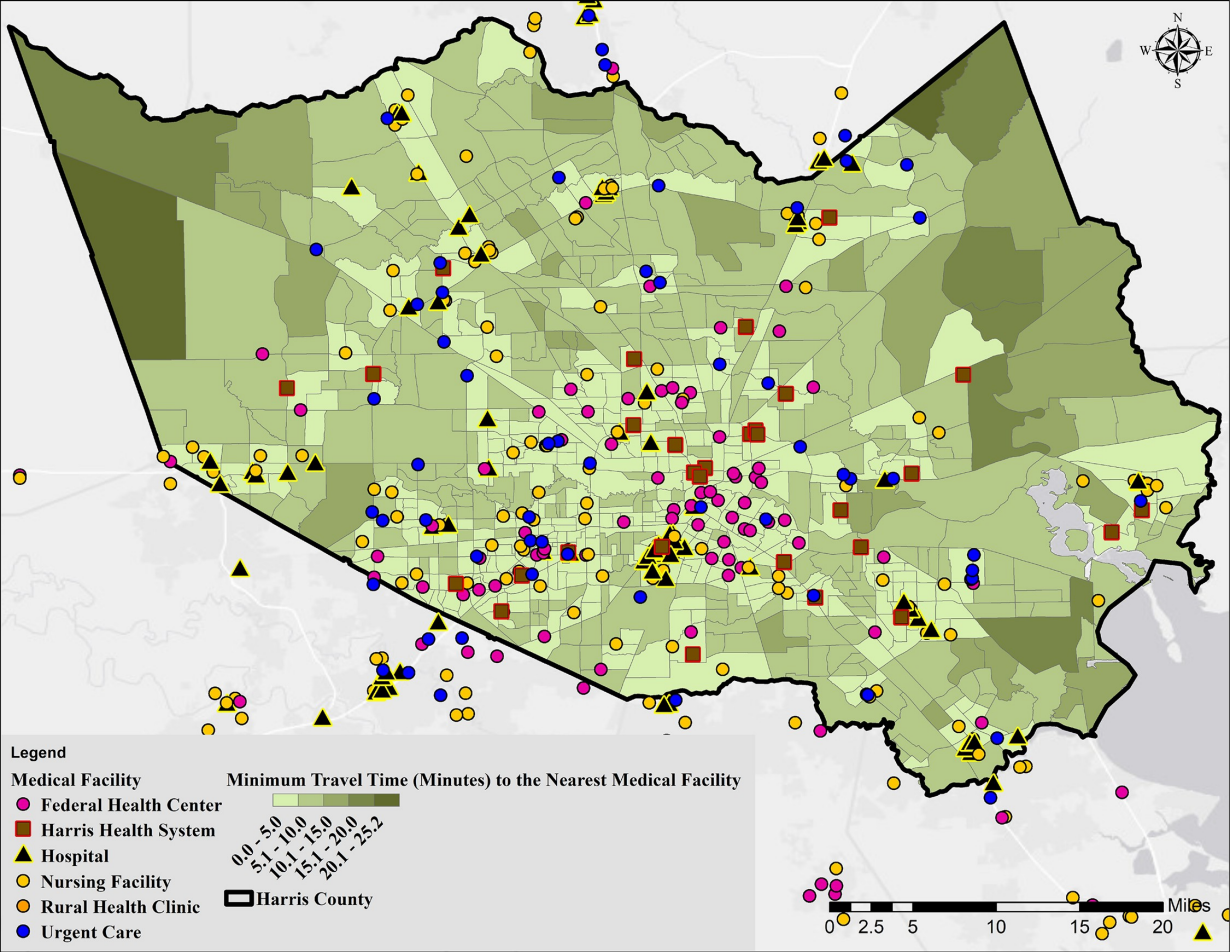
Map showing the distance from the centroid of census tracts to the nearest medical facility.

S2 Table in S1 File provides a summary of statistics for all of the 46 variables used in the study. Among the 46 variables, maps are only presented for those that were not based on publicly available data. Fig 4 shows the locations of medical facilities (all types as described in Methods) within and around Harris County as well as the drive time to the nearest facility for each census tract. The drive time varies from seconds to 25.23 minutes in the no traffic condition, with a median of 4.74 minutes (S2 Table in S1 File). As can be seen from Fig 4, people who live in areas located farther away from the center of the County, especially in the western and northeastern parts, have a longer drive time to a medical care facility. This longer drive time becomes even more critical if an individual does not have a personal car and needs to use the less than a fully functional transportation system. The travel time is even longer to facilities managed by the Harris Health system (typically used by individuals with no insurance or documentation). From a planning standpoint, Fig 4 below, when combined with vulnerabilities, can be used to drive decisions related to the establishment of field hospitals during periods of widespread transmission. Importantly, the data can be used to develop a more holistic response plan directing persons with various severity or symptoms of the disease to different types of medical intervention facilities.

**Fig 5:**
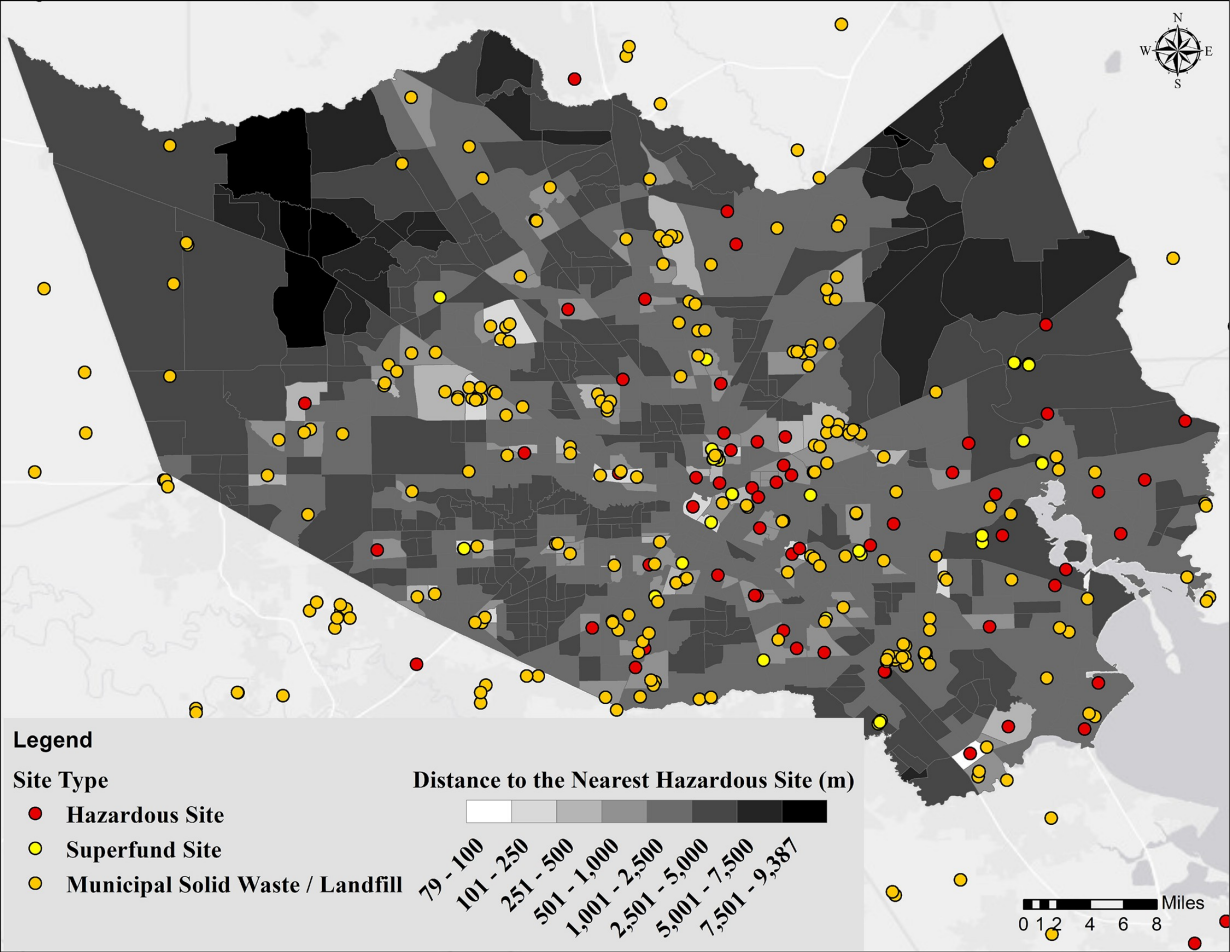
Map showing the distance from the centroid of census tracts to the nearest hazardous site.

The distance to and location of hazardous sites (Superfund sites, landfills, and industrial hazards) are shown in Fig 5. The distance ranges from 79 to 9,386 m with a median of 2,105 m. The hazardous sites are spread over the entire County but are more concentrated closer to the industrial areas (see Fig 1), water bodies (Houston Ship Channel (HSC), and Galveston Bay (GB)). The tracts in less developed areas have the longest distance to a hazardous site indicating a higher potential vulnerability to pollution in the more developed parts of Harris County.

S1-S3 Figs in S1 File show the median concentration of ozone, NO_2_, and PM_2.5_ for each tract in addition to the number of times during 2000–2019 that a monitoring station exceeded the EPA standard. It should be noted that the number of stations and, consequently, the geospatial coverage was significantly lower for NO_2_ and PM_2.5_ when compared to ozone. Stations with the highest number of exceedances of EPA standards for all three measures are located near industrial areas (see Fig 1). While the central parts of the County showed the highest concentration of NO_2_ and PM_2.5_, ozone concentrations were highest closer to the industrial areas. The higher levels of NO_2_ in central parts of Harris County could be attributed to emissions from mobile sources that are more abundant in downtown Houston [52]. The observed pattern for ozone is a result of industrial activities, the ozone-NO_2_ relationship, and the wind pattern in Houston [53, 54]. In the case of PM_2.5_, the higher concentrations in Harris County have been associated with regional aerosols, biomass burning, and gasoline combustion [55] that are higher in the central part of the County. The median concentrations for ozone, NO_2_, and PM_2.5_ were 21.24 ppb, 8.32 ppm, and 9.98 μg/m^3^, respectively, for the period of 2000–2019. What is interesting to note is the fact that the three variables have different spatial distributions thereby indicating potentially more important involvement in COVID-19 based on recent research showing increased vulnerability due to PM_2.5_ pollution in COVID-19 patients [56] and CDC’s indication that “people with moderate to severe asthma may be at higher risk of getting very sick from COVID-19.”

**Fig 6:**
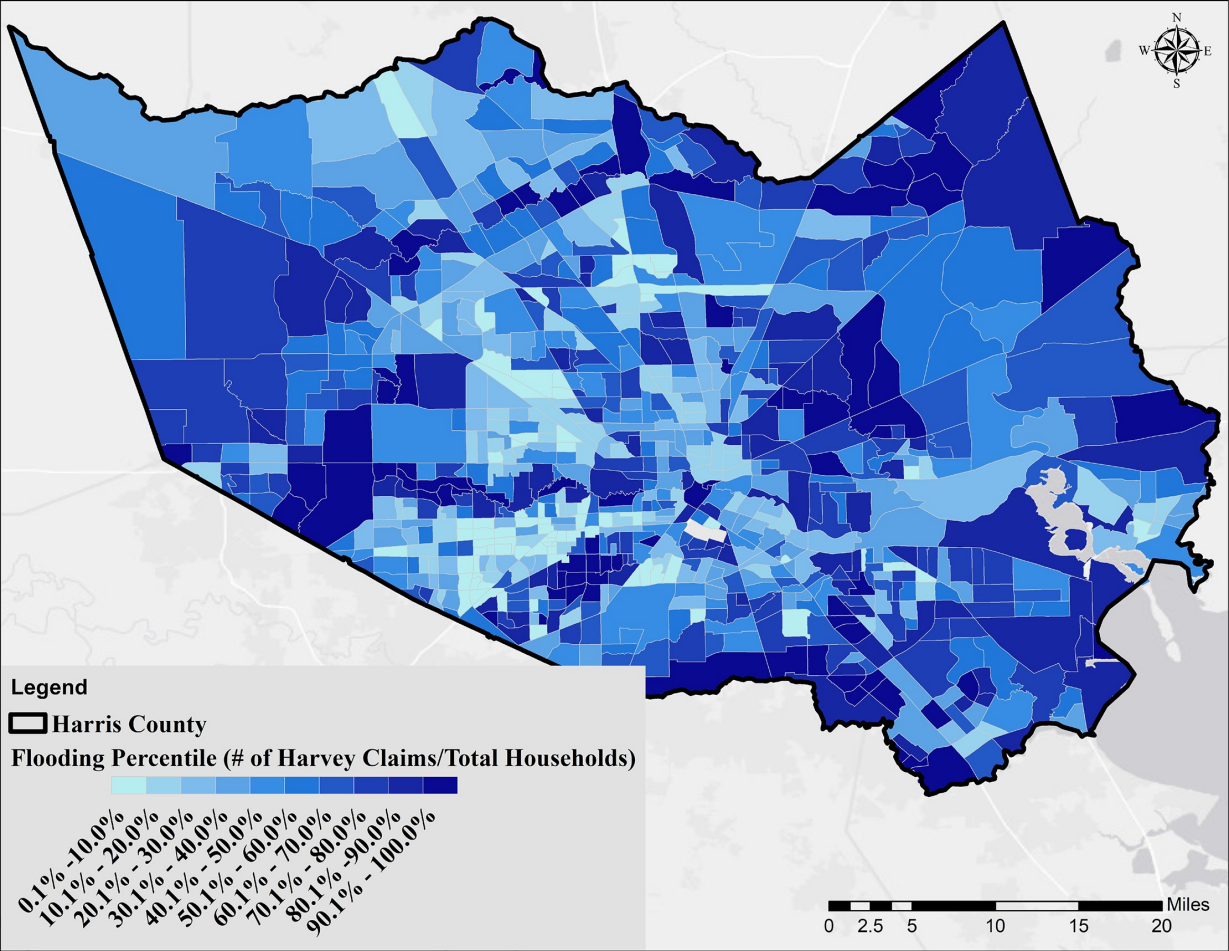
Flooding vulnerability based on the number of households that filed damage claims to the Federal Emergency Management Agency after Hurricane Harvey.

Contaminant discharge events (S4 Fig in S1 File) and the second environmental variable representing dry cleaners, petroleum storage tanks, and IHWCA sites (S5 Fig in S1 File) were substantially higher in industrial areas close to the HSC and GB: La Porte, Baytown, Deer Park, and Channelview, with the number of events as high as 1,449 (2000-present). The median was 12 events across all census tracts. Fig 6 shows the percentile of flooding across Harris County based on the filed claims to FEMA. Areas closer to the bayous/streams and flood control dams showed a higher vulnerability. A similar distribution was observed using the geospatial inundation modeling approach (S6 Fig in S1 File). Finally, S7 Fig in S1 File shows the distribution of educated persons in Harris County, defined as the ratio of age 25 years and over with a high school diploma or higher degree to the total population for each census tract.

### Vulnerability in census tracts

The developed illustrative maps, data and tabulated findings in this work can be directly used by decision-makers to make quantitative comparisons that fit their needs. The results are illustrated here using generalized descriptive language based on the geospatial visualization in order to identify the overall areas of the County with the most vulnerable population. It is noted that using the same methodology and set of variables, except for Category 4 that could be customized based on location, similar tables and maps could be created for any location of interest. For instance, Category 4 could be defined as being vulnerable to drought, wildfire, tornados, earthquakes, and so on. Such vulnerability originates from the fact that i) in the aftermath of each of these natural hazards, the individuals who were impacted may have severe socio-economic-health issues, and ii) a compound event where any of these hazards overlap with the pandemic could substantially amplify the effect of each of the hazards.

**Fig 7:**
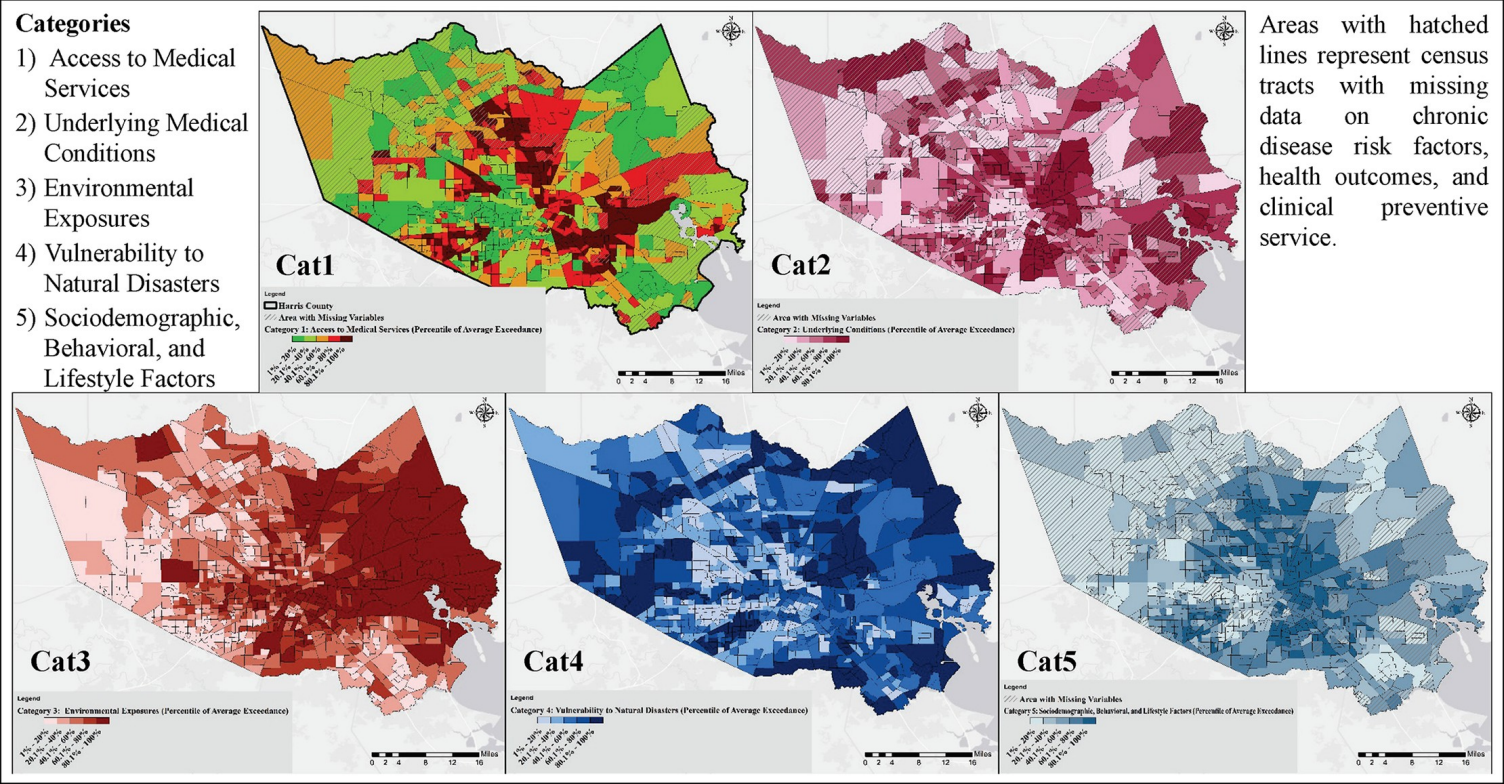
The average exceedance for variables in the 5 categories. Averaged exceedance values are reported in percentiles for the purpose of comparison among tracts.

**Fig 8:**
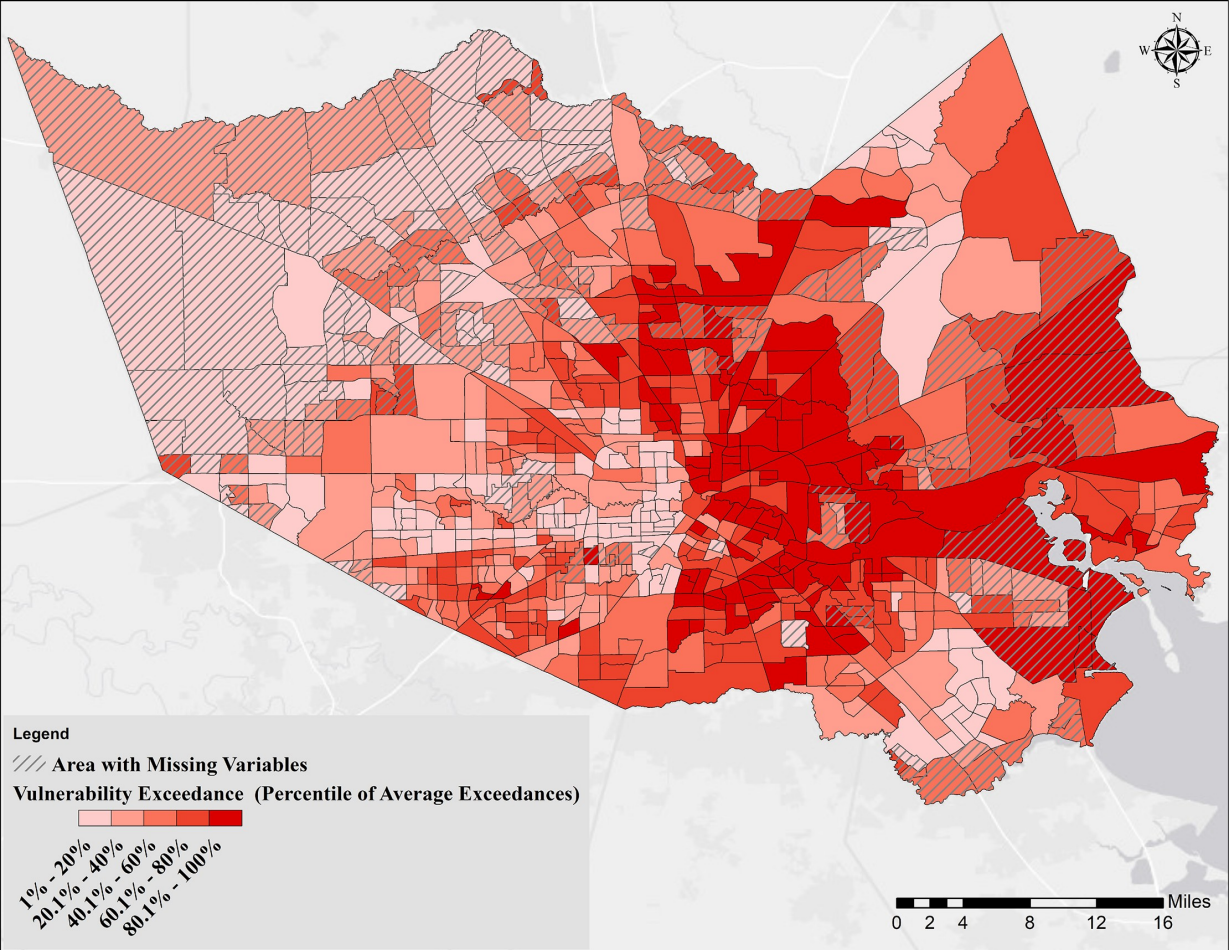
Overall vulnerability based on determinants in all 5 categories. Areas with hatched lines represent census tracts with missing data on chronic disease risk factors, health outcomes, and clinical preventive services [46].

Fig 7 represents the average exceedance for variables within Category 1 through Category 5 (values are reported in percentiles for the purpose of comparison among tracts), respectively, and Fig 8 shows the overall vulnerability for all variables in the five categories. The developed tool can be used to examine each tract individually and compare their vulnerability with other tracts. The final cluster centers for different variables for each category (K-means method) are represented in the S3 Table in S1 File. S8-S12 Figs in S1 File show the class of each tract (i.e., high/severe, average, and low) for Category 1 through Category 5, respectively. The results in all categories were similar to the exceedance methods, validating the choice of methodology. The overall vulnerability generated by the K-means methods led to a very similar map (S13 Fig in S1 File) to the exceedance approach (Fig 8).

**Fig 9:**
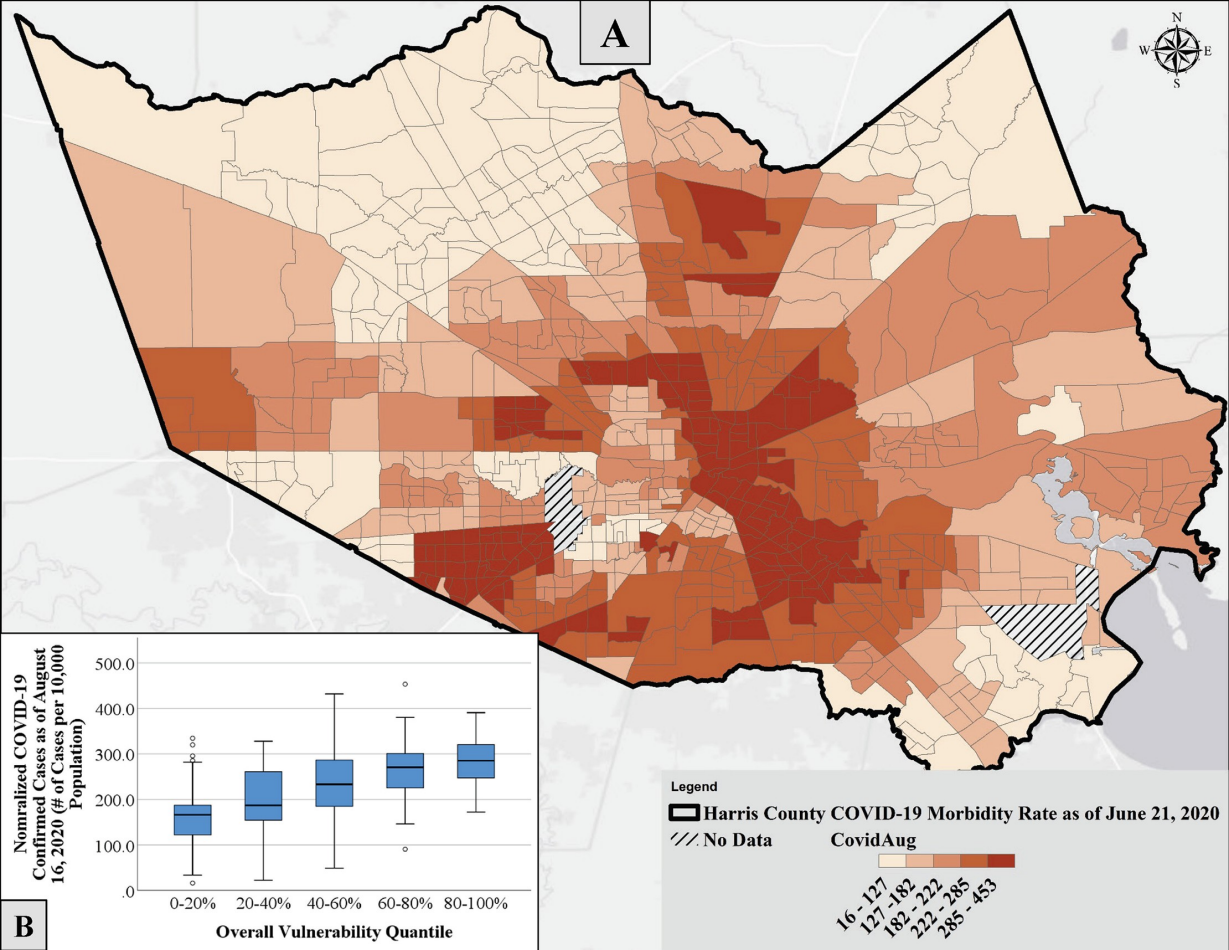
A) Geospatial variability in the normalized COVID-19 confirmed cases in Harris County as of August 16, 2020, at the census tract level. The data was converted from a zip code level database, and B) distribution of normalized cases among tracts with different levels (quantiles) of overall vulnerabilities.

Fig 9A shows the normalized total number of confirmed COVID-19 cases as of August 16, 2020, in Harris County at the census tract level. It should be noted that the original dataset at the zip code level was converted to census tract level using ArcMap. Since the conversion was completed using an area-based weighted average, the maximum number of normalized cases is different from the original dataset; 453 and 212 confirmed COVID-19 cases per 10,000 persons for the converted and original datasets, respectively. By comparing Figs 9 and 7, a lot of similarities in the geospatial distribution of vulnerability (Fig 7) and morbidity (Fig 9) can be observed. Spearman’s Correlation analysis showed a significant and relatively strong correlation (P-value = 2.6E-51, ρ = 0.570) between the normalized number of cases and the overall vulnerability exceedance rate. While all categories showed significant correlations with the normalized cases, Cat 5 followed by Cat 1 showed the most strong relationships with a correlation coefficient of 0.616 (P-value = 7.2E-62) and 0.566 (P-value = 2.3E-50), respectively. The correlation coefficients for Cat 2, Cat 3, and Cat 4 were 0.39 (P-value = 1.6E-22), 0.344 (P-value = 3.4E-23), and 0.203 (P-value = 9.3E-09), respectively. Fig 9B depicts the distribution of normalized cases among tracts with different levels (quantiles) of overall vulnerabilities. The general increasing trend in the box plots shown in Fig 9B supported by visual similarities and, most importantly, by statistical tests validates the performance and reliability of the developed tool. To further validate the developed tool, the vulnerability to natural disasters was estimated for Nueces County, TX, during Hurricane Harvey using the FEMA data. A significant and relatively strong correlation (P-value = 0.017, ρ = 0.471) was observed between the number of COVID-19 confirmed cases (as of August 16, 2020, S14A Fig in S1 File), and the total number of Harvey claims at the zip code level (S14B Fig in S1 File).

Overall, the vulnerabilities associated with each category exhibited varying geospatial distributions with some commonality (i.e., some census tracts had elevated vulnerabilities in each category). Category 1, 2, and 5 vulnerabilities shown in Fig 7 (access to medical services, underlying medical conditions, and sociodemographic, respectively) indicated a similar finding; the most severe vulnerability can be observed in areas with the least favorable conditions represented by the three categories (lowest income, lower education levels, less insurance coverage, unhealthy diet and lifestyles, and more underlying medical conditions). Category 3 (environmental exposures) showed a spatially declining gradient from east to west with some hotspots around downtown Houston. This gradient could be explained by the presence of the majority of industrial activities in the eastern part of Harris County, and worse air quality near downtown Houston. Category 4 (vulnerability to natural disasters) showed the highest risk in the vicinity of major bayous/streams in the County, as discussed before.

Looking at Fig 8, it could be concluded that the most vulnerable persons to COVID-19 in Harris County, are living in the eastern part of the County, specifically areas next to the HSC and GB, and areas identified as opportunity zones [57]. The residents in these neighborhoods are individuals belonging to disadvantaged or historically marginalized groups, are exposed to several chemicals (with industrial sources), and subject to flooding both from rainfall and storm surge (such as what was experienced during Hurricane Ike in 2008). The relationship between race and ethnicity and physiological vulnerability to COVID-19 is beyond the scope of this paper since the medical data are insufficient at this time to complete such an analysis. However, due to disparities in the distribution of wealth, welfare, and services in the US, African-American and Hispanic populations are more likely to have lower levels of health, education, and income. The relative ratios of African-American and Hispanic populations across Harris County are shown in S15, S16 Figs in S1 File, respectively. By looking at Figs 8, 9A, and S15, S16 Figs in S1 File, it could be concluded that the majority of tracts with the highest ratio of African-American and Hispanic population are located in areas with the highest overall vulnerability and normalized COVID-19 cases. Individuals living in the western and southeastern fringe of the County are least vulnerable. However, it is noted that the underlying medical condition data were not available for those tracts. It is also noted that individuals in those areas, if infected, especially in the western fringe, will have significantly limited access to medical services compared to the other parts of the County.

### Vulnerable population estimates in Harris County

For each category, the total population and the distribution of population in two age intervals, 45–65 (the age group with the highest number of reported COVID-19 cases), and +65 (the age group with the highest mortality rate), over different percentiles (from low to high with regards to the severity of conditions within each category) is shown in Table 2. Using the vulnerability findings presented above for Harris County (Fig 7, and yellow highlighted values in Table 2); a total of 59,307; 98,702; 78,723; 105,431; and 59,624 seniors (+65 years), who are at most risk of COVID-19 mortality, are living in areas with the highest vulnerability in Category 1 through 5, respectively. Considering the fact that Harris County is prone to flooding and the hurricane season is in progress from May through the end of November, a potential hurricane combined with the COVID-19 pandemic could lead to a compound natural disaster event affecting significant numbers of senior citizens as shown in Table 2. Decision-makers, to prepare for the worst-case pandemic scenario and occurrence of a hurricane, in particular, could use the numbers in Category 1 and 4 for planning response and recovery measures that take into account flooding and increased vulnerability to COVID-19. For overall vulnerability (Fig 8 and cyan highlights in Table 2), a total of 722,357 persons (~17% of the population of the County) including 171,403 with ages between 45–65 (~4% of the total population of Harris County), and 76,719 seniors (~2% of the population of the County and 10.6% of total identified vulnerable population), are at a higher overall risk. As of August 16, 2020, 92,944 confirmed COVID-19 cases were reported in Harris County [37], which is 12.7% of the estimated vulnerable people identified in this study. Among those, ~14,600 (15.7% of total confirmed cases) are seniors compared to the 10.6% estimated by the model. The disagreement between the model estimates and reported numbers by Harris County [37] could be due to the testing rates. According to [58], Texas is among the states with the lowest testing rates (15.46%), with a higher testing rate among the seniors. It is expected that as time passes, the number of cases increases in the County both because of expanding the testing rates and due to further spread of the pandemic.

**Table 2:** Distribution of the total population, and those of ages between 45 and 65, and above 65 years within different determinant category percentiles of vulnerability in Harris County.

Percentile		0% - 20%	20% - 40%	40% - 60%	60% - 80%	80% - 100%
Category 1	Total Population	760,572	929,535	946,254	892,448	846,173
	45–65 years	212,214	236,982	232,755	210,859	173,524
	> 65 years	113,479	103,052	96,236	80,253	59,307
Category 2	Total Population	1,107,588	923,925	922,086	786,298	635,085
	45–65 years	234,744	222,531	230,052	204,197	174,810
	> 65 years	71,517	86,353	98,296	97,459	98,702
Category 3	Total Population	1,016,883	914,746	830,344	795,215	817,794
	45–65 years	269,504	224,015	202,769	182,543	187,503
	> 65 years	108,117	95,234	90,642	79,611	78,723
Category 4	Total Population	745,835	831,232	970,245	928,682	898,988
	45–65 years	163,690	199,625	235,323	230,867	236,829
	> 65 years	68,795	86,866	95,116	96,119	105,431
Category 5	Total Population	1,057,398	867,297	909,292	824,148	716,847
	45–65 years	292,819	220,506	212,651	188,267	152,091
	> 65 years	123,228	101,213	93,823	74,439	59,624
Overall Vulnerability	Total Population	998,996	927,584	906,212	819,833	722,357
	45–65 years	249,385	241,270	213,158	191,118	171,403
	> 65 years	97,587	108,035	88,496	81,490	76,719

See Fig 7 for Categories 1 through 5 and Fig 8 for Overall Vulnerability geospatial distributions.

### Limitations and future work

The limitation in data availability should be considered when interpreting these results. First, data was lacking for some of the variables for a number of census tracts in Harris County; the CDC’s 500 Cities dataset [46] for example is limited to the City of Houston and does not include the rest of the County. The analysis findings might vary if a Houston-specific dataset is used. However, the downside is that the approach would not incorporate important county-level considerations. Second, the use of the COVID-19 dataset up to current dates in terms of manuscript preparation (August 16, 2020 in this study) and not having the full benefit of the comprehensive infection dataset prior to the availability of vaccinations or effective treatments could affect the results and, consequently, interpretations of the findings. Future work could include new variables such as occupational exposure to COVID-19, and taking into account populations that have the ability to work from home and those that do not. This may be a key component of vulnerability, especially in the context of Stay Home Orders issued by various States.

## Conclusions

In this novel project, we develop a planning tool that can help identify populations at higher risk of infection, morbidity, and mortality from COVID-19 at the census tract level. These findings can guide the allocation of scarce resources, and thus, are relevant to policymakers at all levels of government. Effectively using the results from the planning tool to inform actions could mean the difference between suppressing the virus and allowing it to re-emerge. In comparison, it is noted that studies that map the geospatial spread of coronavirus from Wuhan to neighboring communities are starting to emerge [59, 60], and similar efforts need to be launched in the U.S. The application of geospatial methods to case data enables significantly more rigor in understanding the confluence of various factors that jointly increase vulnerabilities and reduce resilience to COVID-19 spread, impact, re-emergence in new hot spots or on a seasonal basis. While geospatial indices exist [30, 32], they are not tailored to the unique features of this virus. Lastly, the findings from this study enable public health departments to efficiently and equitably allocate resources, including preventive, therapeutic, and rehabilitative measures. For example, our results can guide where testing and vaccine distribution sites should be located, which communities would benefit most from policies that mandate physical distancing and masks, which hospitals need additional support because they are at risk for exceeding their capacities, and where contact tracers should target their efforts. Reports of long wait times for testing and uncertainty about where to invest resources [61] suggest that policy makers lack the data they need to make these decisions.

## Supporting information

S1 File (DOCX)
