# Supplementary material for: Assessing COVID-19 risk, vulnerability and infection prevalence in communities
Source: PLoS One. 2020 Oct 29;15(10):e0241166. doi: 10.1371/journal.pone.0241166 (PMC7595430; doi:10.1371/journal.pone.0241166)
Supplement: S1 File — (DOCX) [file pone.0241166.s001.docx]

**Tables:**

**S1 Table. Rotated component matrix from the Principal Component Analysis. Varimax with Kaiser Normalization was chosen as the rotation method. Highlighted values show values greater than 0.5 or the maximum among the components.**

| Variable | Component | | | | |
| --- | --- | --- | --- | --- | --- |
|  | 1 | 2 | 3 | 4 | 5 |
| Access to HBP medications | -0.16 | -0.91 | 0.03 | 0.15 | 0.16 |
| Age above 50 | -0.22 | 0.87 | 0.01 | -0.11 | 0.00 |
| Age above 60 | -0.22 | 0.88 | -0.03 | -0.01 | -0.01 |
| Age above 70 | -0.17 | 0.88 | -0.08 | 0.05 | 0.02 |
| Age above 80 | -0.16 | 0.75 | -0.09 | 0.16 | 0.07 |
| Arthritis | 0.53 | 0.80 | 0.00 | -0.13 | -0.08 |
| Asthma | 0.90 | -0.02 | 0.07 | -0.06 | -0.33 |
| At least one disability | 0.46 | 0.55 | 0.18 | -0.02 | -0.20 |
| Below the poverty line | 0.81 | -0.16 | 0.07 | 0.24 | 0.04 |
| Binge drinking | -0.81 | -0.44 | 0.11 | 0.05 | 0.04 |
| Cancer | -0.30 | 0.89 | -0.11 | -0.09 | -0.03 |
| Cholesterol | 0.61 | 0.66 | -0.03 | -0.11 | 0.31 |
| Cholesterol screening | 0.66 | -0.65 | 0.05 | 0.16 | 0.25 |
| Chronic heart disease | 0.73 | 0.63 | 0.09 | -0.02 | 0.11 |
| Chronic kidney disease | 0.85 | 0.48 | 0.13 | 0.03 | -0.02 |
| COPD | 0.90 | 0.34 | 0.03 | -0.02 | 0.03 |
| Current smoker | 0.94 | -0.27 | 0.04 | 0.02 | 0.02 |
| Diabetes | 0.91 | 0.35 | 0.13 | -0.05 | -0.01 |
| Distance to a hazardous site | 0.03 | -0.12 | 0.28 | 0.01 | 0.06 |
| Drive time to a medical facility | -0.10 | -0.08 | -0.12 | -0.60 | -0.14 |
| Education beyond high school diploma | 0.76 | -0.24 | 0.08 | 0.00 | 0.48 |
| Fecal Occult blood test, Sigmiudiscioy, or Colonoscopy | 0.82 | -0.40 | 0.04 | 0.08 | 0.35 |
| FEMA Harvey claims ratio | -0.30 | 0.24 | 0.01 | -0.60 | -0.02 |
| Harvey inundation ratio | -0.06 | 0.01 | -0.03 | -0.50 | 0.06 |
| HBP | 0.75 | 0.60 | 0.06 | -0.11 | -0.23 |
| Household density | -0.12 | -0.26 | -0.16 | 0.70 | -0.10 |
| Insurance coverage | 0.73 | -0.37 | -0.13 | 0.14 | 0.36 |
| Living alone | -0.21 | 0.05 | 0.26 | 0.61 | -0.38 |
| Low sleep quality | 0.90 | -0.24 | 0.06 | -0.03 | -0.25 |
| Mammography | 0.74 | -0.26 | -0.04 | 0.13 | 0.56 |
| Median age (year ) | -0.38 | 0.76 | 0.03 | -0.06 | -0.02 |
| No physical activity | 0.96 | -0.12 | 0.05 | 0.02 | 0.22 |
| NO_2_ concentration | -0.02 | 0.14 | 0.85 | 0.31 | -0.01 |
| Number of dry cleaners, petroleum storage tanks, and IHWCA sites | 0.07 | -0.11 | 0.36 | -0.28 | 0.21 |
| Number of hazardous releases | 0.05 | 0.03 | 0.01 | -0.16 | 0.42 |
| Obesity | 0.96 | -0.08 | 0.15 | -0.04 | -0.05 |
| Older men up to date on core clinical preventive services | 0.86 | -0.27 | 0.15 | -0.02 | -0.01 |
| Older women up to date on core clinical preventive services | 0.92 | -0.24 | 0.15 | 0.08 | 0.14 |
| Ozone concentration | -0.13 | 0.02 | -0.88 | 0.01 | 0.06 |
| Pap Smear | 0.87 | -0.23 | -0.03 | 0.14 | 0.30 |
| PM_2.5_ concentration | 0.19 | 0.05 | 0.80 | -0.01 | -0.12 |
| Poor Mental condition | 0.92 | -0.30 | 0.05 | 0.03 | 0.16 |
| Poor Physical condition | 0.96 | 0.04 | 0.08 | 0.02 | 0.22 |
| Stroke | 0.84 | 0.46 | 0.13 | -0.01 | -0.20 |
| Visit doctor for routine checkup | 0.06 | -0.76 | 0.03 | 0.20 | 0.58 |
| Visit to dentist or dental clinic | 0.94 | -0.27 | 0.08 | 0.06 | 0.14 |

**S2 Table. Descriptive summary statistics for all 46 variables. Median and median absolute deviation are reported instead of mean and standard deviation because none of the datasets were normally distributed**

| Variable | N | Minimum | Maximum | Median | Median Absolute Deviation |
| --- | --- | --- | --- | --- | --- |
| Household density (number of houses per 1,000 sq. m) | 786 | 0.00 | 8.04 | 0.64 | 0.28 |
| Drive time to a medical facility (minutes) | 786 | 0.00 | 25.23 | 4.74 | 1.57 |
| Access to HBP medications (%) | 584 | 25.90 | 85.10 | 72.50 | 6.10 |
| Cholesterol screening (%) | 584 | 39.10 | 88.80 | 67.80 | 8.90 |
| Insurance coverage (%) | 786 | 42.00 | 100.00 | 80.00 | 9.00 |
| Visit doctor for routine checkup (%) | 584 | 54.80 | 80.40 | 67.30 | 4.25 |
| Visit to dentist or dental clinic (%) | 584 | 25.20 | 82.50 | 51.85 | 15.95 |
| Mammography (%) | 584 | 61.70 | 81.20 | 74.00 | 5.00 |
| Pap Smear (%) | 584 | 56.20 | 86.40 | 75.60 | 7.20 |
| Fecal Occult blood test, Sigmiudiscioy, or Colonoscopy (%) | 584 | 28.70 | 74.20 | 54.05 | 11.55 |
| Older men up to date on core clinical preventive services (%) | 584 | 0.00 | 39.90 | 25.70 | 6.10 |
| Older women up to date on core clinical preventive services (%) | 584 | 0.00 | 45.40 | 28.05 | 7.65 |
| Arthritis (%) | 584 | 4.00 | 30.90 | 18.55 | 4.30 |
| Asthma (%) | 584 | 6.30 | 14.60 | 8.70 | 1.30 |
| HBP (%) | 584 | 9.80 | 52.00 | 29.50 | 5.80 |
| Cancer (%) | 584 | 0.80 | 10.80 | 4.60 | 1.40 |
| Cholesterol (%) | 584 | 13.20 | 48.50 | 38.05 | 4.65 |
| Chronic kidney disease (%) | 584 | 0.80 | 6.00 | 2.70 | 0.70 |
| COPD (%) | 584 | 1.70 | 14.80 | 5.70 | 2.00 |
| Chronic heart disease (%) | 584 | 0.80 | 11.40 | 5.60 | 1.90 |
| Diabetes (%) | 584 | 2.10 | 25.50 | 11.90 | 4.40 |
| Poor Mental condition (%) | 584 | 6.10 | 21.80 | 12.60 | 3.60 |
| Poor Physical condition (%) | 584 | 4.70 | 26.40 | 13.20 | 5.00 |
| Stroke (%) | 584 | 0.50 | 10.00 | 2.80 | 1.10 |
| At least one disability (%) | 786 | 1.00 | 100.00 | 9.00 | 2.00 |
| Median age (year ) | 786 | 0.00 | 60.40 | 33.55 | 3.35 |
| Age above 50 (%) | 786 | 0.00 | 66.00 | 27.00 | 6.00 |
| Age above 60 (%) | 786 | 0.00 | 51.00 | 15.00 | 4.00 |
| Age above 70 (%) | 786 | 0.00 | 29.00 | 6.00 | 2.00 |
| Age above 80 (%) | 786 | 0.00 | 14.00 | 2.00 | 1.00 |
| Distance to a hazardous site (m) | 786 | 79.3 | 9386.8 | 2105.4 | 858.5 |
| Number of hazardous releases (unitless) | 786 | 1 | 1469 | 12 | 6 |
| Number of dry cleaners, petroleum storage tanks, and IHWCA sites | 786 | 0 | 60 | 4 | 2 |
| Ozone concentration (ppb) | 786 | 17.57 | 26.57 | 21.24 | 1.35 |
| NO_2_ concentration (ppm) | 786 | 4.62 | 16.20 | 8.32 | 1.41 |
| PM_2.5_ concentration (µg/m^3^) | 786 | 8.24 | 11.70 | 9.98 | 0.37 |
| FEMA Harvey claims ratio (%) | 786 | 0.00 | 0.37 | 0.04 | 0.02 |
| Harvey inundation ratio (%) | 786 | 0.00 | 0.99 | 0.12 | 0.12 |
| Binge drinking (%) | 584 | 8.10 | 27.80 | 15.70 | 2.60 |
| Current smoker (%) | 584 | 7.00 | 34.40 | 18.10 | 5.65 |
| No physical activity (%) | 584 | 15.40 | 51.40 | 33.20 | 9.60 |
| Obesity (%) | 584 | 18.30 | 51.50 | 34.60 | 7.80 |
| Low sleep quality (%) | 584 | 24.10 | 49.00 | 37.00 | 5.50 |
| Education beyond high school diploma (%) | 786 | 30.77 | 100.00 | 83.02 | 12.00 |
| Below the poverty line (%) | 786 | 0.00 | 100.00 | 14.00 | 8.00 |
| Living alone (%) | 786 | 0.00 | 100.00 | 24.00 | 8.00 |

**S3 Table: Results of the K-Means analysis: final cluster centers for different variables at each category**

| Category | Variable | Cluster | | |
| --- | --- | --- | --- | --- |
|  |  | 1 | 2 | 3 |
| Category 1 | Household density (number of houses per 1,000 sq. m) | 0.89 | 0.75 | 0.98 |
|  | Drive time to a medical facility (minutes) | 4.31 | 4.85 | 5.07 |
|  | Access to HBP medicine (%) | 29.46 | 26.91 | 26.68 |
|  | Cholesterol screening (%) | 40.06 | 30.87 | 21.66 |
|  | Insurance coverage (%) | 66.99 | 78.02 | 91.23 |
|  | Visit doctor for routine checkup (%) | 34.92 | 31.03 | 30.62 |
|  | Visit to dentist or dental clinic (%) | 61.18 | 46.81 | 27.76 |
|  | Mammography (%) | 31.13 | 25.49 | 21.71 |
|  | Pap Smear (%) | 31.38 | 23.68 | 17.39 |
|  | Fecal Occult blood test, Sigmiudiscioy, or Colonoscopy (%) | 56.65 | 44.54 | 33.32 |
|  | Older men up to date on core clinical preventive services (%) | 78.60 | 73.81 | 65.73 |
|  | Older women up to date on core clinical preventive services (%) | 78.00 | 71.07 | 61.53 |
| Category 2 | Arthritis (%) | 26.48 | 19.24 | 14.97 |
|  | Asthma (%) | 10.9 | 8.91 | 7.85 |
|  | HBP (%) | 44.68 | 31 | 24.14 |
|  | Cancer (%) | 5.58 | 4.7 | 4.67 |
|  | Cholesterol (%) | 42.76 | 39.01 | 32.16 |
|  | Chronic kidney disease (%) | 4.18 | 2.94 | 2.06 |
|  | COPM (%) | 9.02 | 6.32 | 3.77 |
|  | Chronic heart disease (%) | 8.82 | 6.11 | 3.78 |
|  | Diabetes (%) | 20.68 | 13.5 | 7.97 |
|  | Mental condition not good (%) | 14.96 | 13.69 | 9.69 |
|  | Physical condition not good (%) | 18.46 | 14.99 | 8.72 |
|  | Stroke (%) | 6.23 | 3.25 | 1.89 |
|  | At least one disability (%) | 16.99 | 9.5 | 7.48 |
|  | Median age (year ) | 35.94 | 32.93 | 34.82 |
|  | Age above 50 (%) | 32.39 | 26.44 | 26.65 |
|  | Age above 60 (%) | 19.88 | 14.86 | 15.25 |
|  | Age above 70 (%) | 9.67 | 6.51 | 6.43 |
|  | Age above 80 (%) | 3.46 | 2.36 | 2.29 |
| Category 3 | Distance to a hazardous site (m) | 1571 | 1451 | 1409 |
|  | Number of hazardous events (unitless) | 1455.91 | 542.81 | 18.8 |
|  | Number of dry cleaners, petroleum storage tanks, and IHWCA sites | 10.55 | 7.67 | 4.55 |
|  | Ozone concentration (ppb) | 23.18 | 21.67 | 21.29 |
|  | NO_2_ concentration (ppm) | 7.52 | 8.95 | 8.82 |
|  | PM_2.5_ concentration (µg/m^3^) | 8.71 | 10.22 | 10.13 |
| Category 4 | FEMA Harvey claims ratio (%) | 11.00 | 5.00 | 3.00 |
|  | Harvey inundation ratio (%) | 75.00 | 36.00 | 6.00 |
| Category 5 | Binge drinking (%) | 18.22 | 16.10 | 13.47 |
|  | Current smoker (%) | 17.02 | 22.63 | 11.10 |
|  | No physical activity (%) | 11.52 | 17.60 | 23.19 |
|  | Obesity (%) | 25.70 | 34.08 | 41.35 |
|  | Low sleep quality (%) | 30.37 | 36.90 | 41.07 |
|  | Education beyond high school diploma (%) | 94.61 | 76.97 | 62.45 |
|  | Below the poverty line (%) | 7.92 | 16.07 | 28.75 |
|  | Living alone (%) | 32.94 | 26.95 | 26.75 |

**Figures:**

**
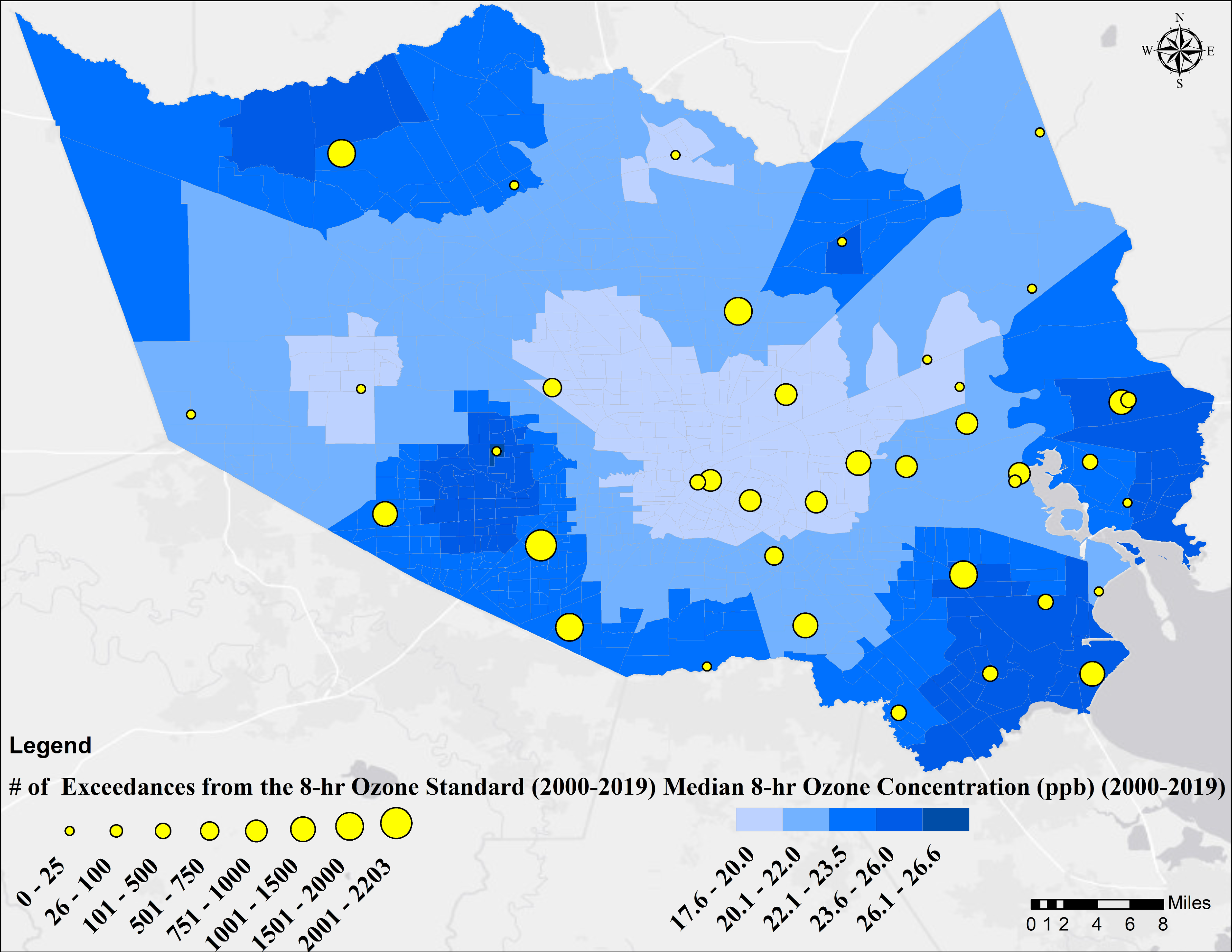
**

**S1 Fig. Map showing the median of averaged 8-hr concentration of Ozone (ppb) in Harris County. The yellow dots show the location of air quality monitoring stations maintained by the TCEQ. The magnitude of the dots shows how many times the averaged 8-hr ozone concentration in a specific station exceeded the 70 ppb standard established by EPA from 2000-2019**

**
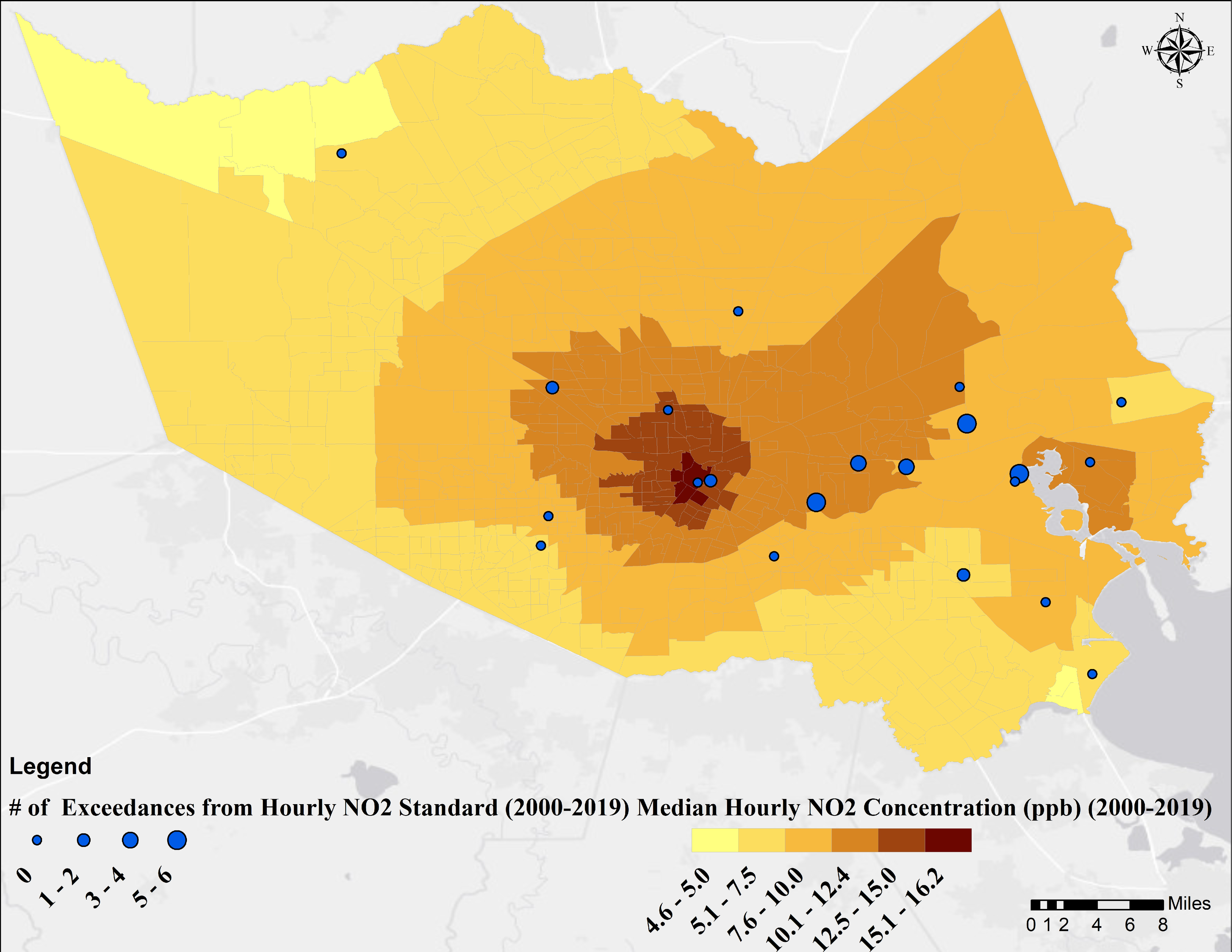
**

**S2 Fig. Map showing the median of hourly concentration of NO_2_ in Harris County. The yellow dots show the location of air quality monitoring stations maintained by TCEQ. The magnitude of the dots shows how many times the hourly NO_2_ concentration in a specific station exceeded the 100 ppb standard established by EPA from 2000-2019**

**
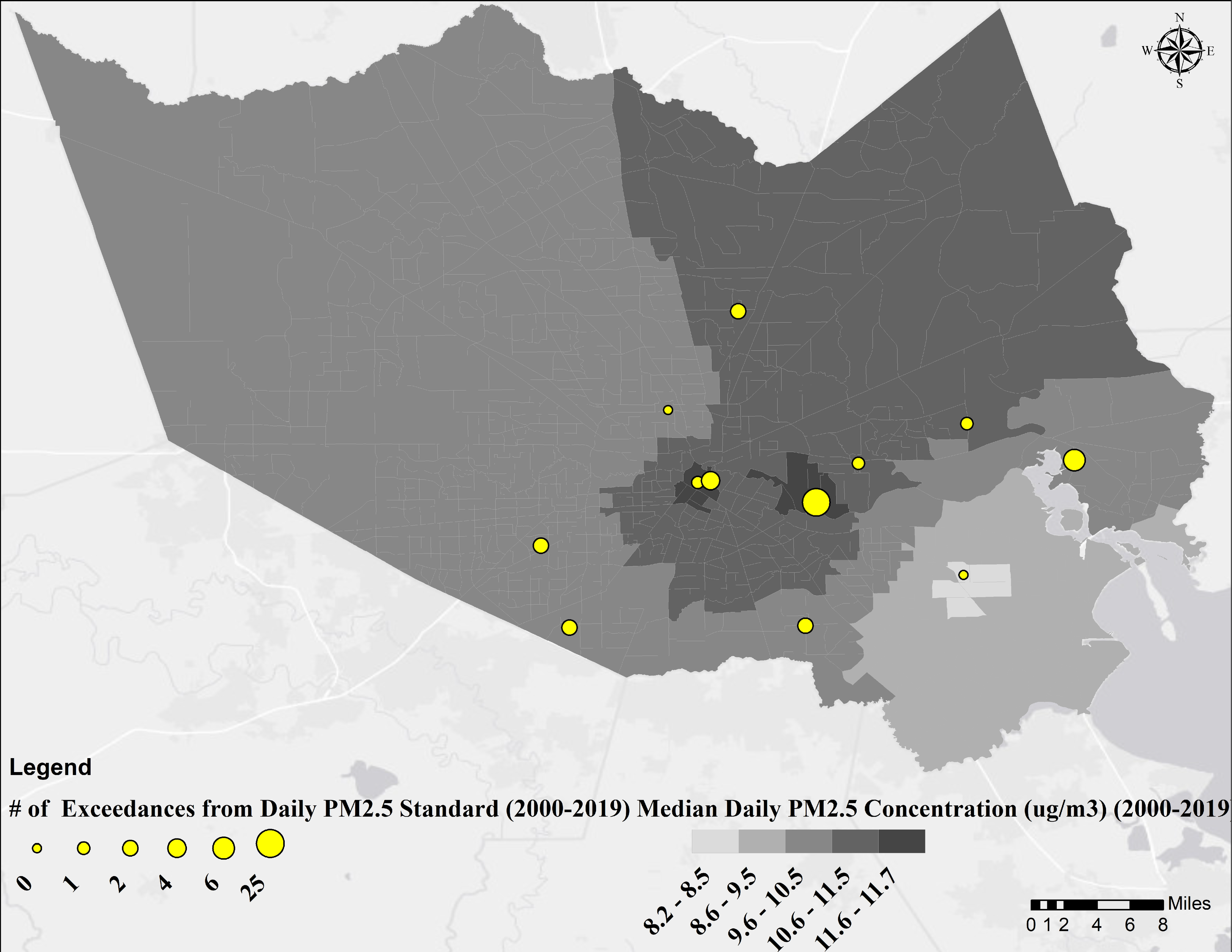
**

**S3 Fig. Map showing the median of averaged daily concentration of PM_2.5_ in Harris County. The yellow dots show the location of air quality monitoring stations maintained by TCEQ. The magnitude of the dots shows how many times the averaged daily PM_2.5_ concentration in a specific station exceeded the 35 µg/m^3^ standard established by EPA from 2000-2019**

**
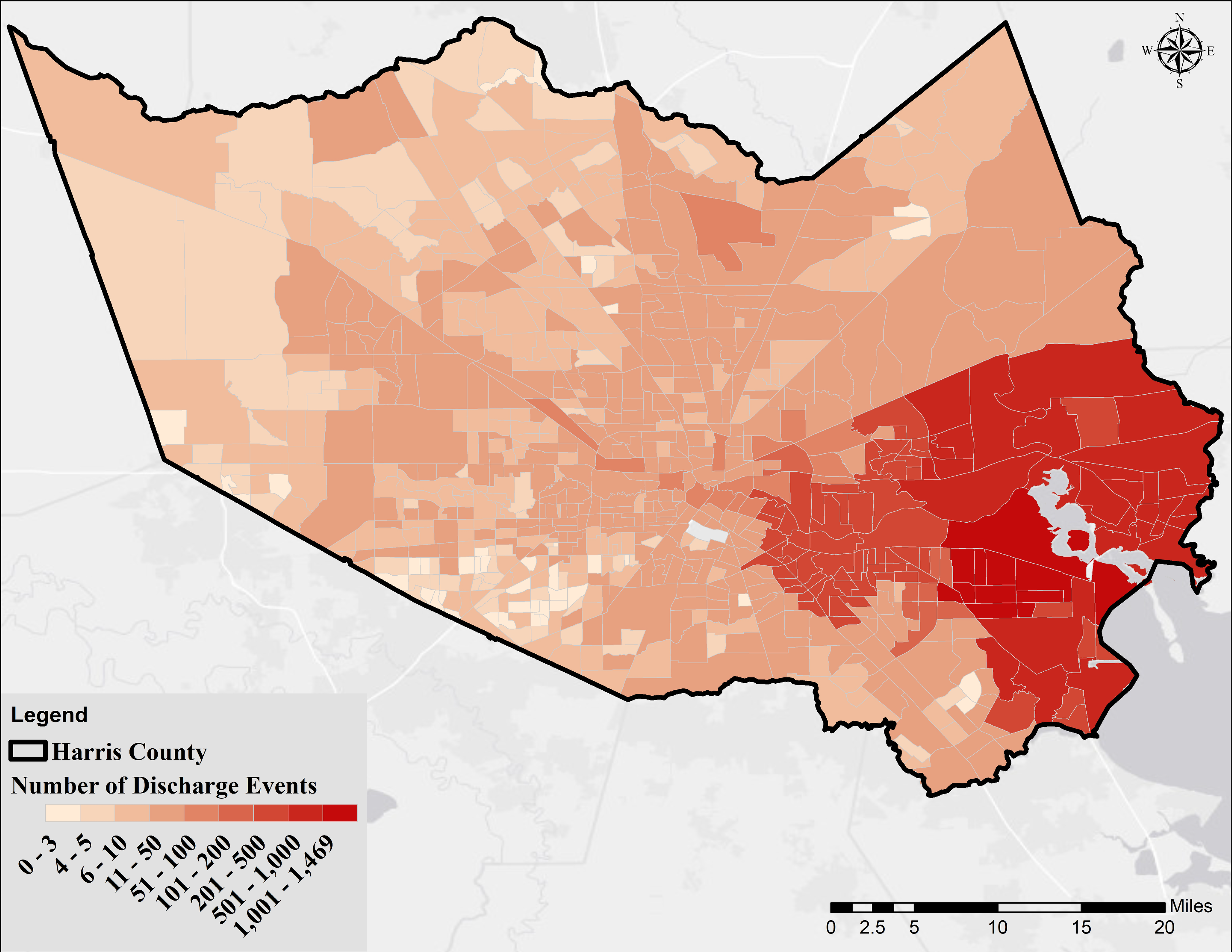
**

**S4 Fig. Map showing the sum of reported environmental release events from 2000-2020 and leaking petroleum tanks within each census tract**

**
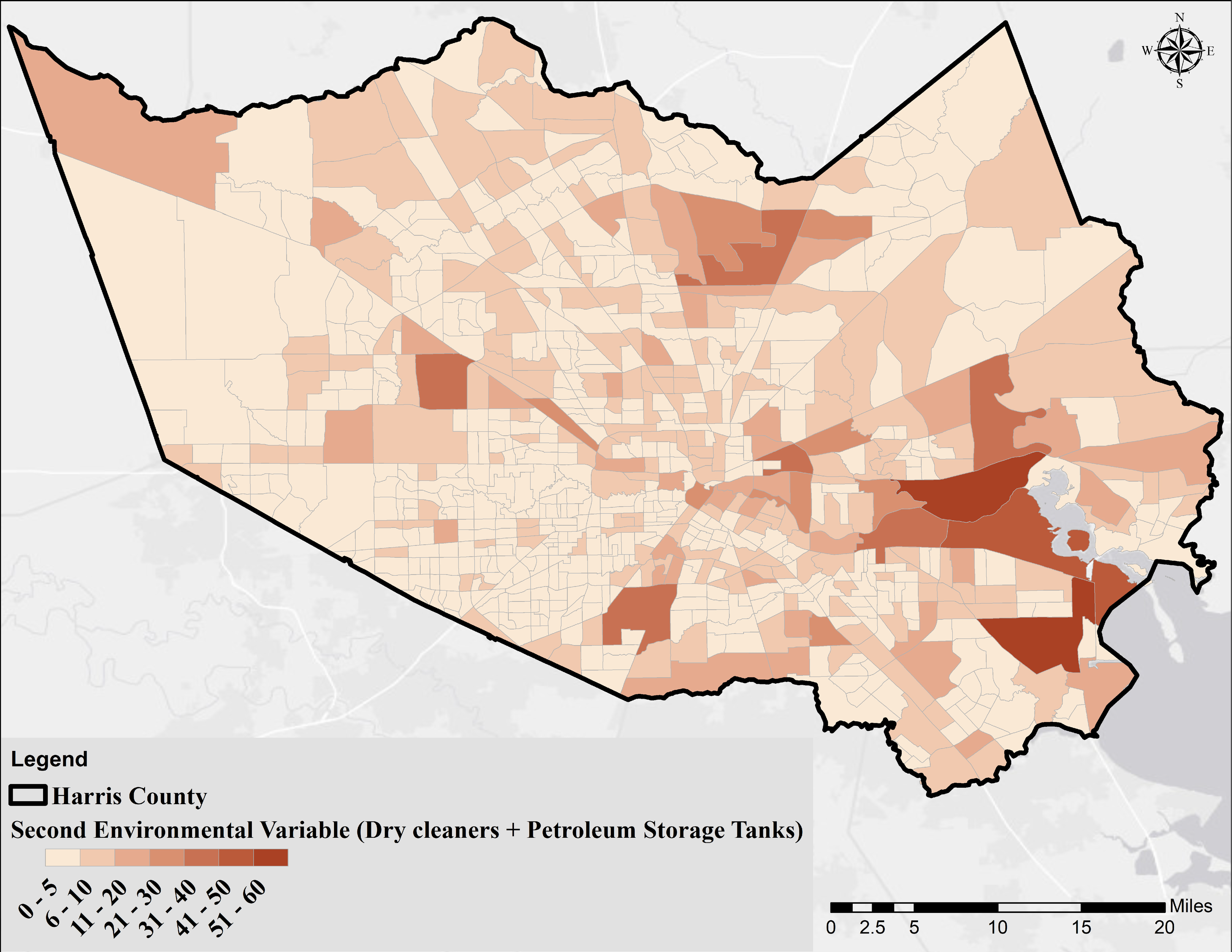
**

**S5 Fig. Map showing the second environmental variable, which is the sum of the number of dry cleaners, petroleum tanks, and sites that are part of an Industrial and Hazardous Waste Corrective Action (IHWCA) program defined by the TCEQ within each census tract**

**
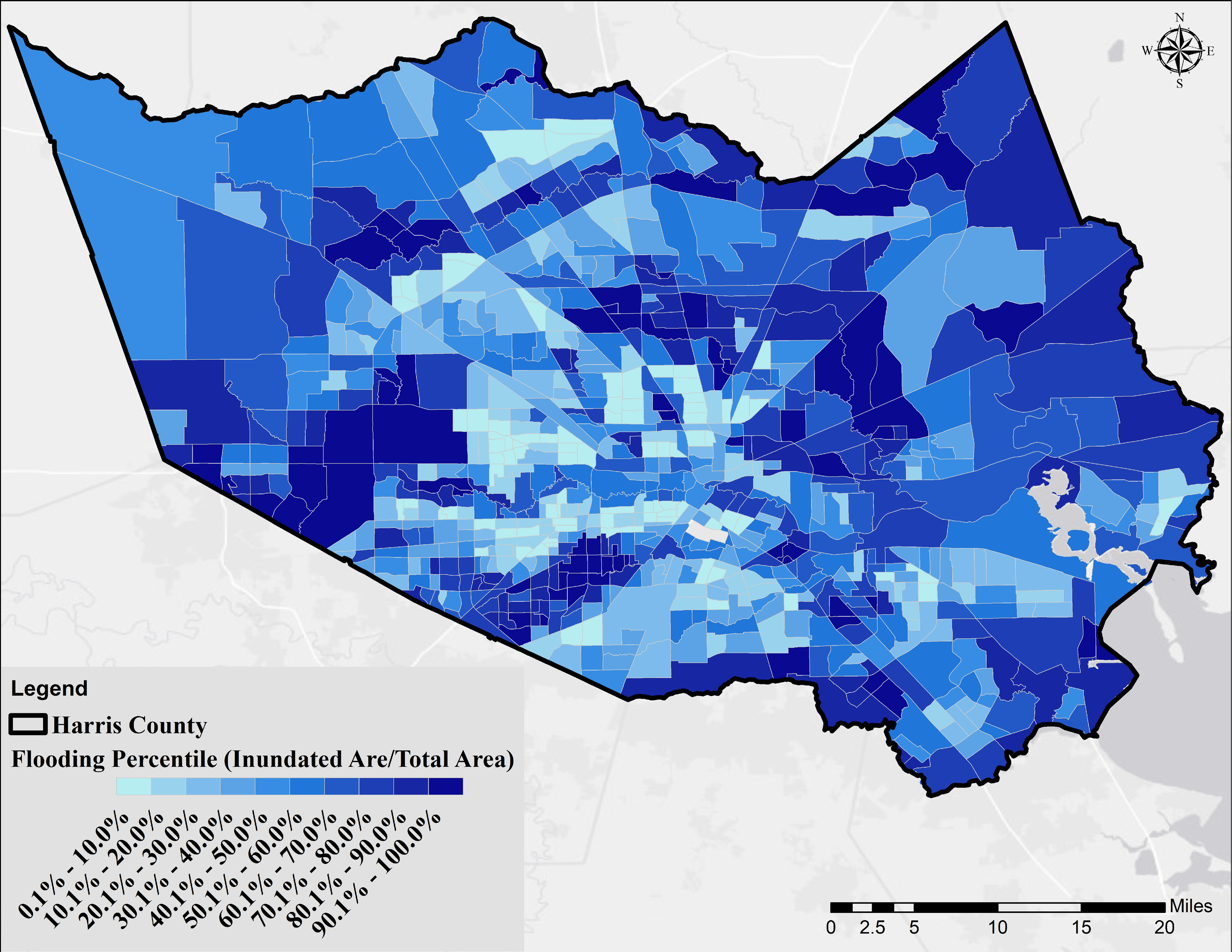
**

**S6 Fig. Map showing flood vulnerability based on the inundation method**

**
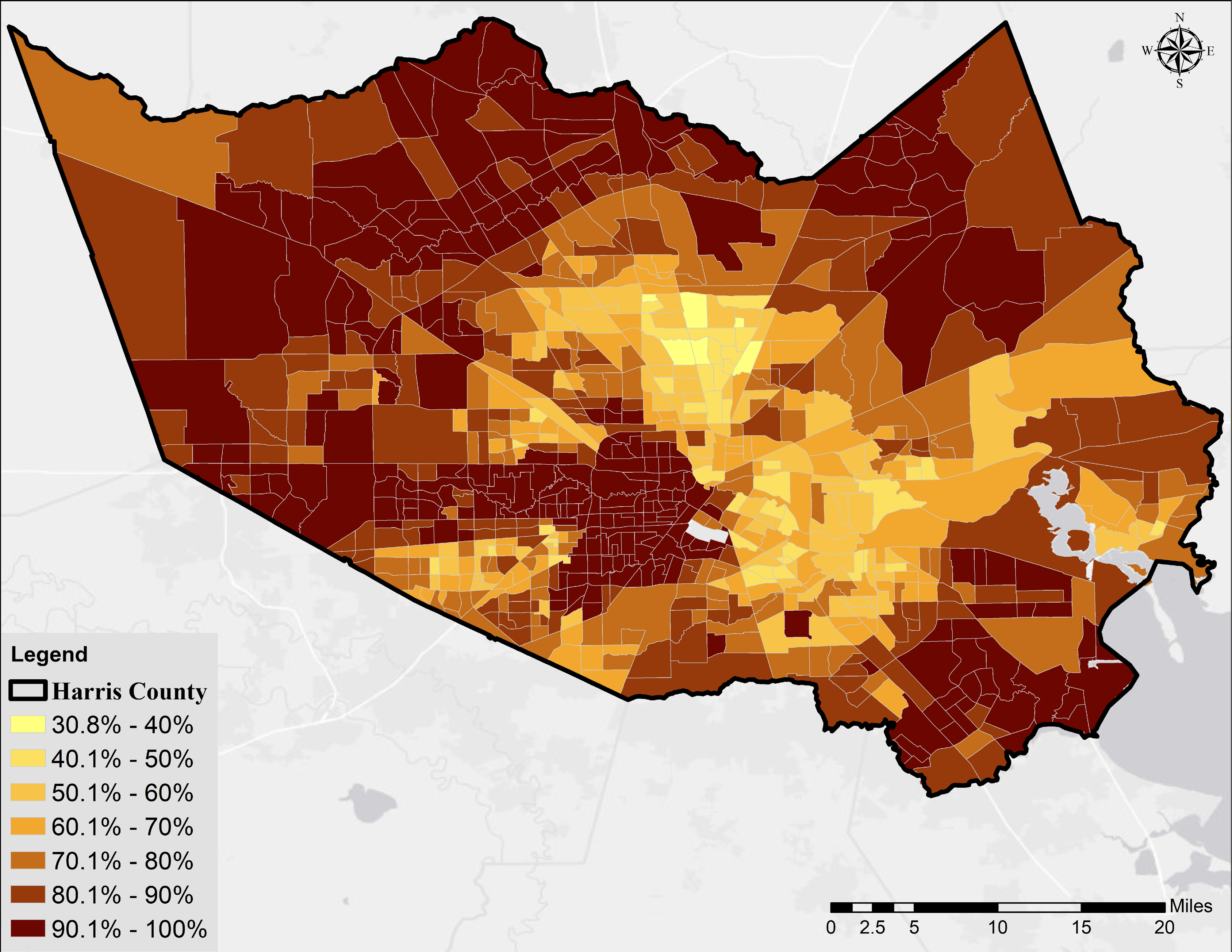
**

**S7 Fig. Map showing the percentage of age 25 years and over to the total population in each census tract with a high school diploma or higher degree**

**
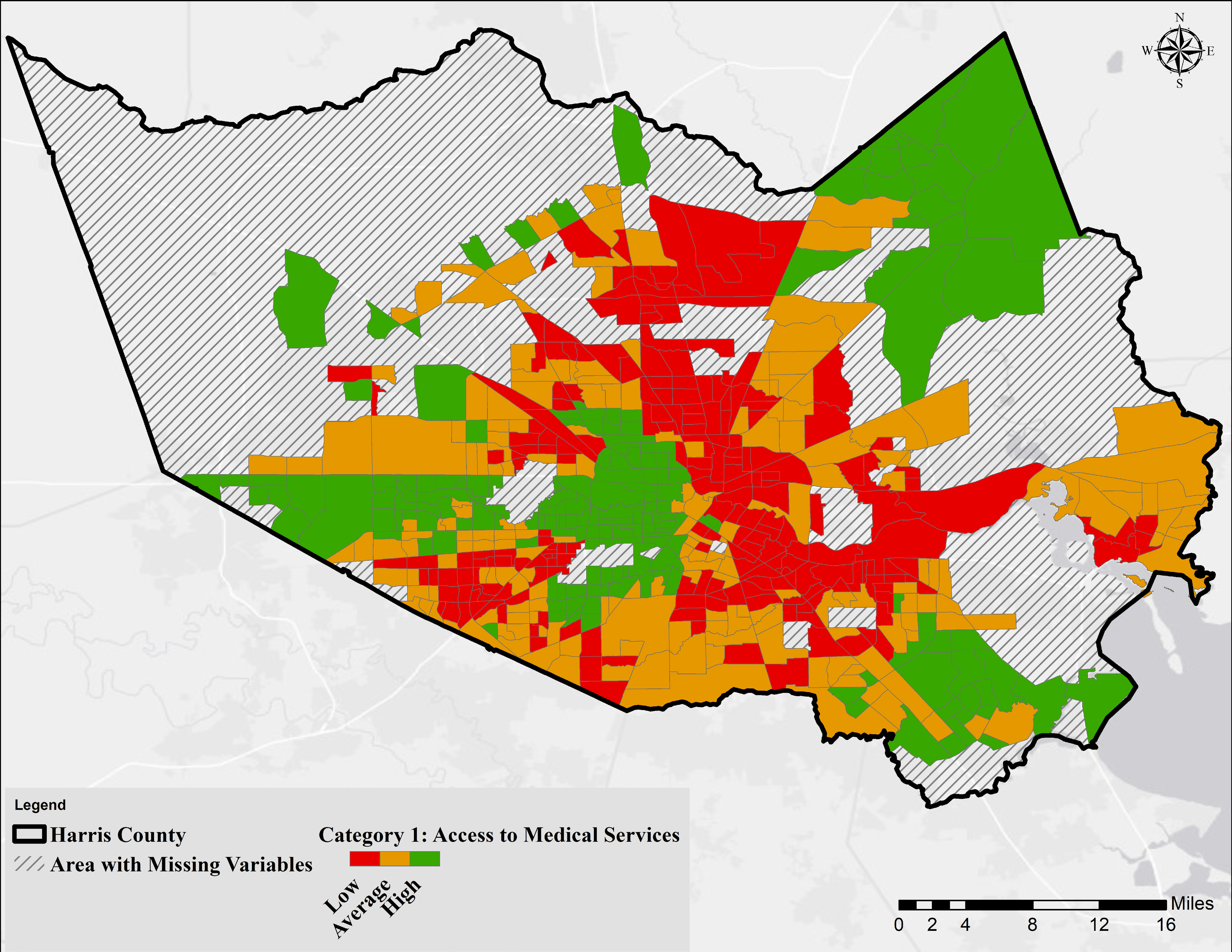
**

**S8 Fig. Results of the K-means cluster analysis. Different colors reflect the belongingness of tracts to the three classes defined in the method for Category 1: Access to Medical (see Table 1 for more details). Areas with hatched lines represent census tracts with missing data on chronic disease risk factors, health outcomes, and clinical preventive service form [1]**

**
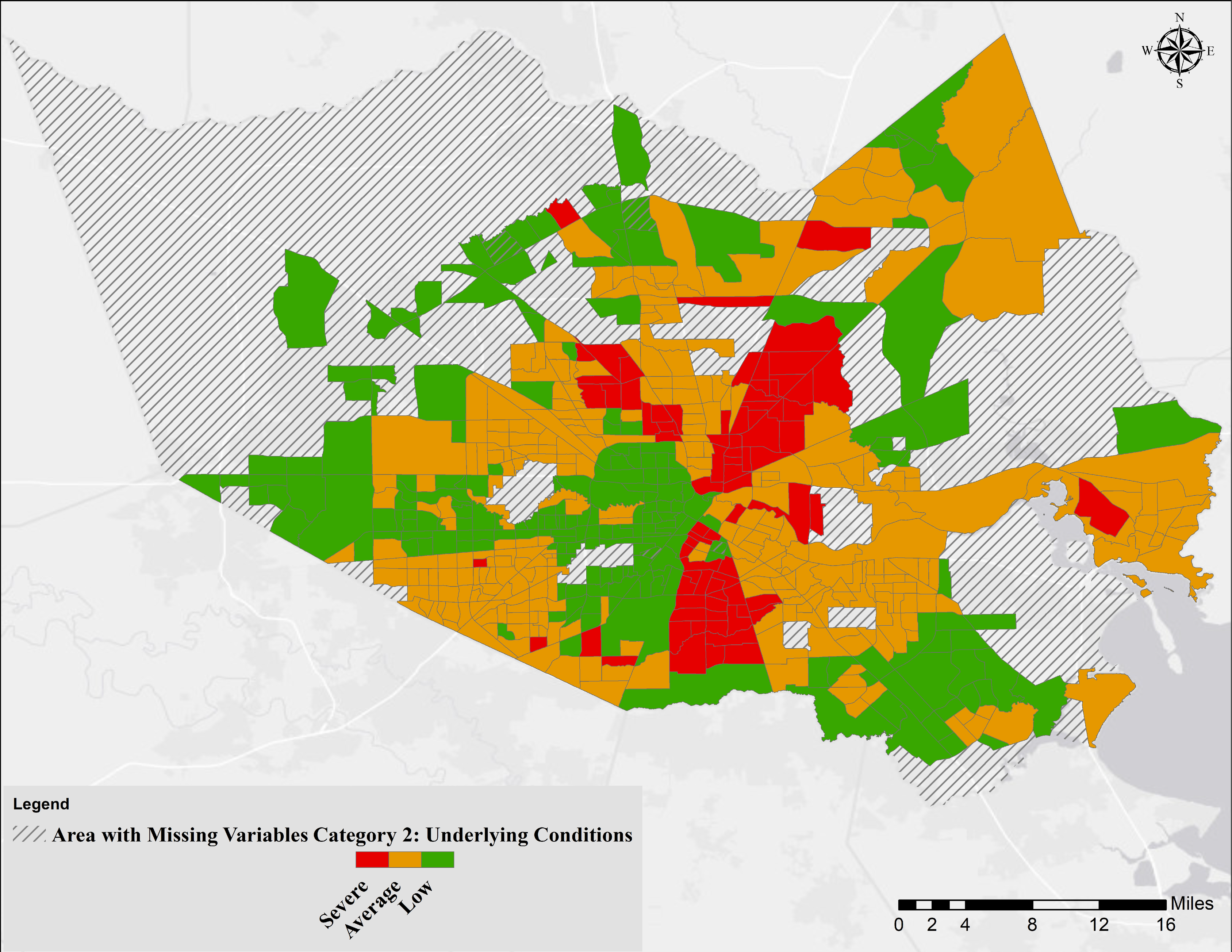
**

**S9 Fig. Results of the K-means cluster analysis. Different colors reflect the belongingness of tracts to the three classes defined in the method for Category 2: Underlying Conditions (see Table 1 for more details). Areas with hatched lines represent census tracts with missing data on chronic disease risk factors, health outcomes, and clinical preventive service form [1]**

**
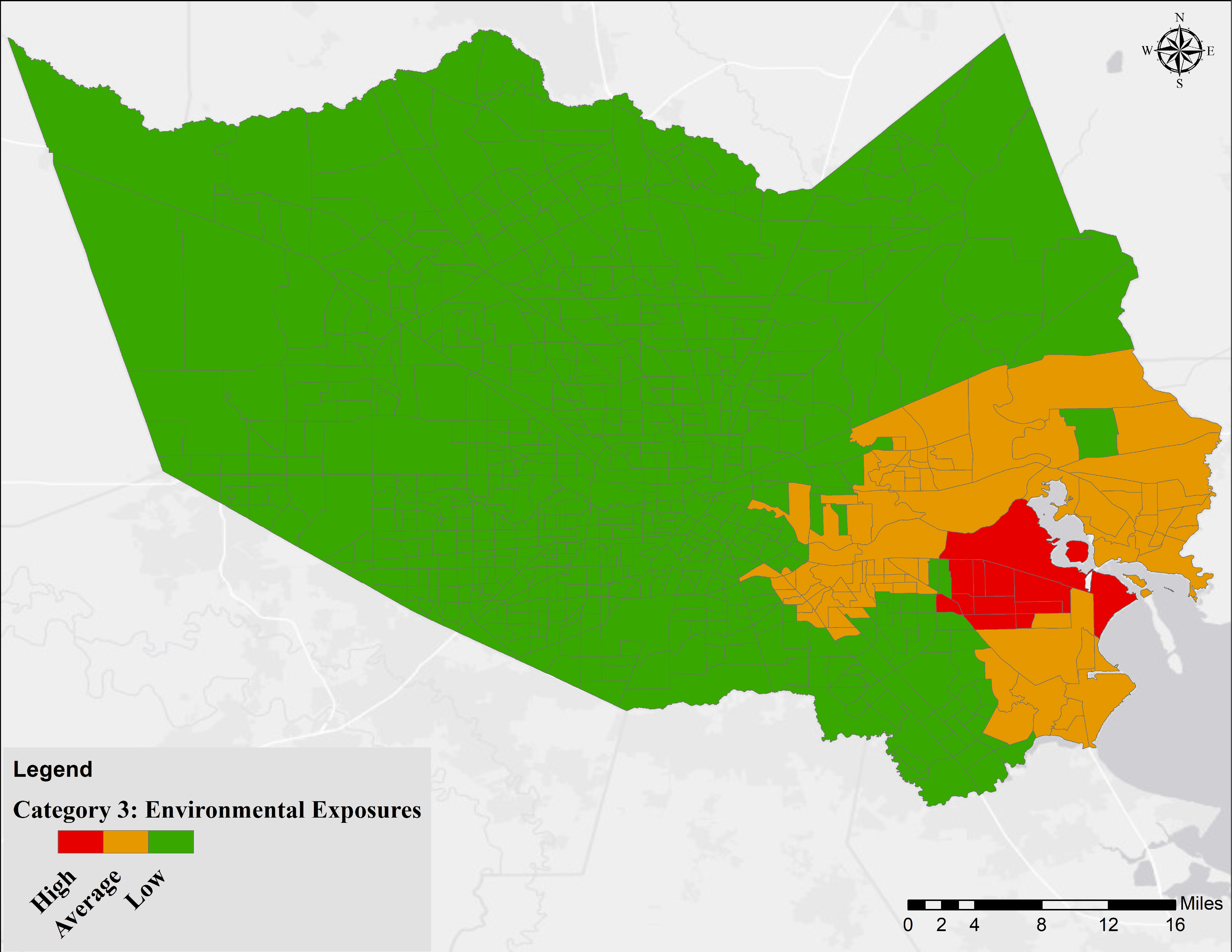
**

**S10 Fig. Results of the K-means cluster analysis. Different colors reflect the belongingness of tracts to the three classes defined in the method for Category 3: Environmental Exposures (see Table 1 for more details)**

**
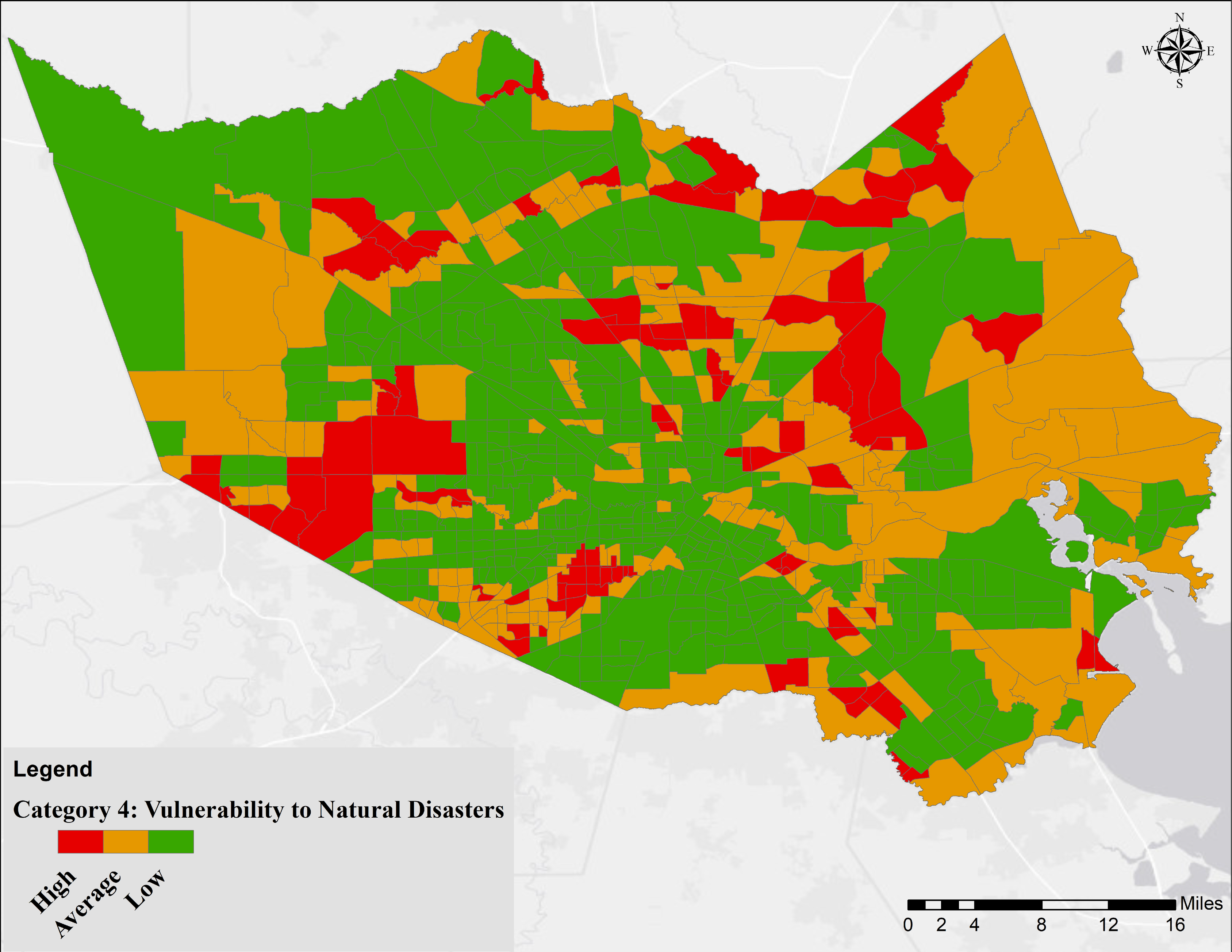
**

**S11 Fig. Results of the K-means cluster analysis. Different colors reflect the belongingness of tracts to the three classes defined in the method for Category 4: Vulnerability to Natural Disasters (see Table 1 for more details)**

**
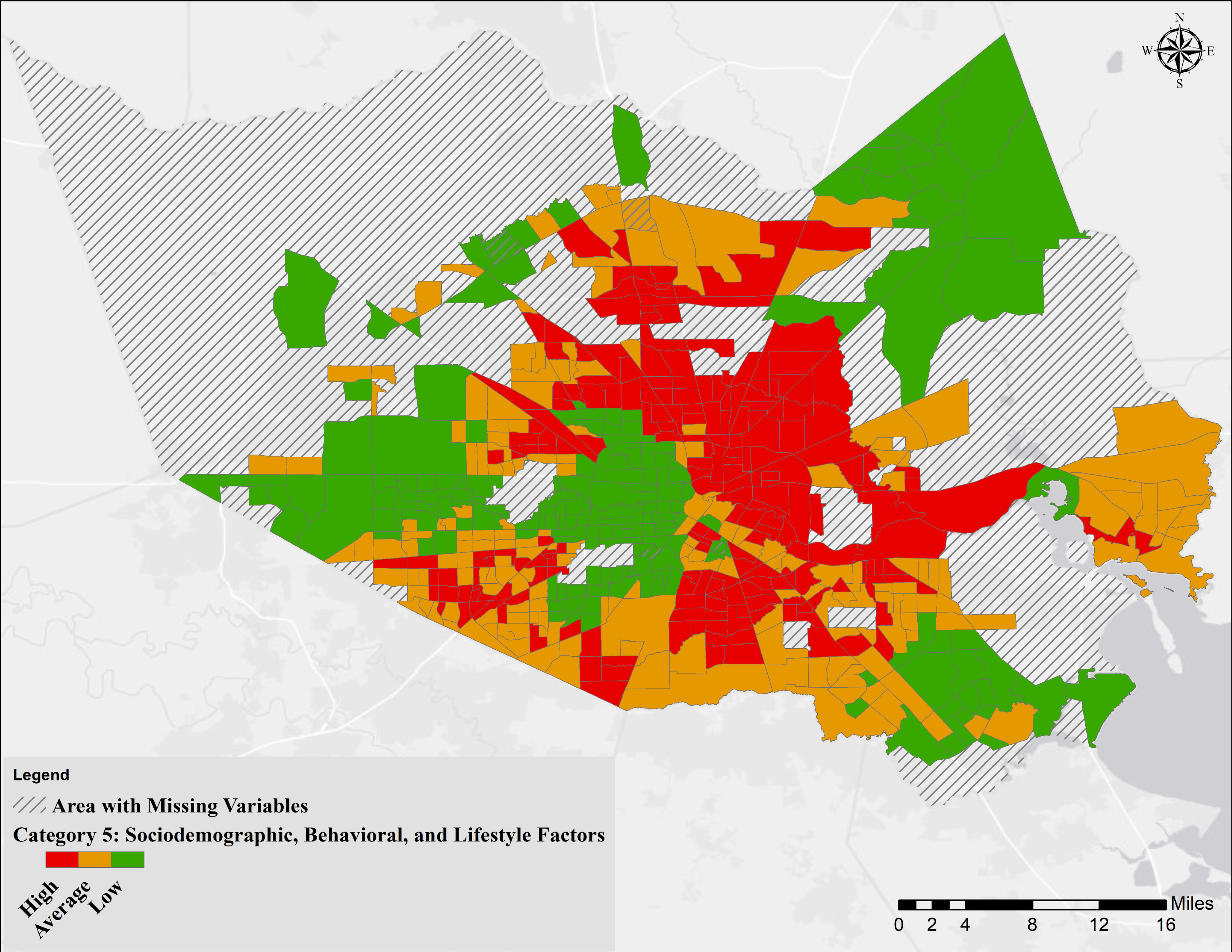
**

**S12 Fig. Results of the K-means cluster analysis. Different colors reflect the belongingness of tracts to the three classes defined in the method for Category 5: Sociodemographic, Behavioral, and Lifestyle Factors (see Table 1 for more details). Areas with hatched lines represent census tracts with missing data on chronic disease risk factors, health outcomes, and clinical preventive service form [1]**

**
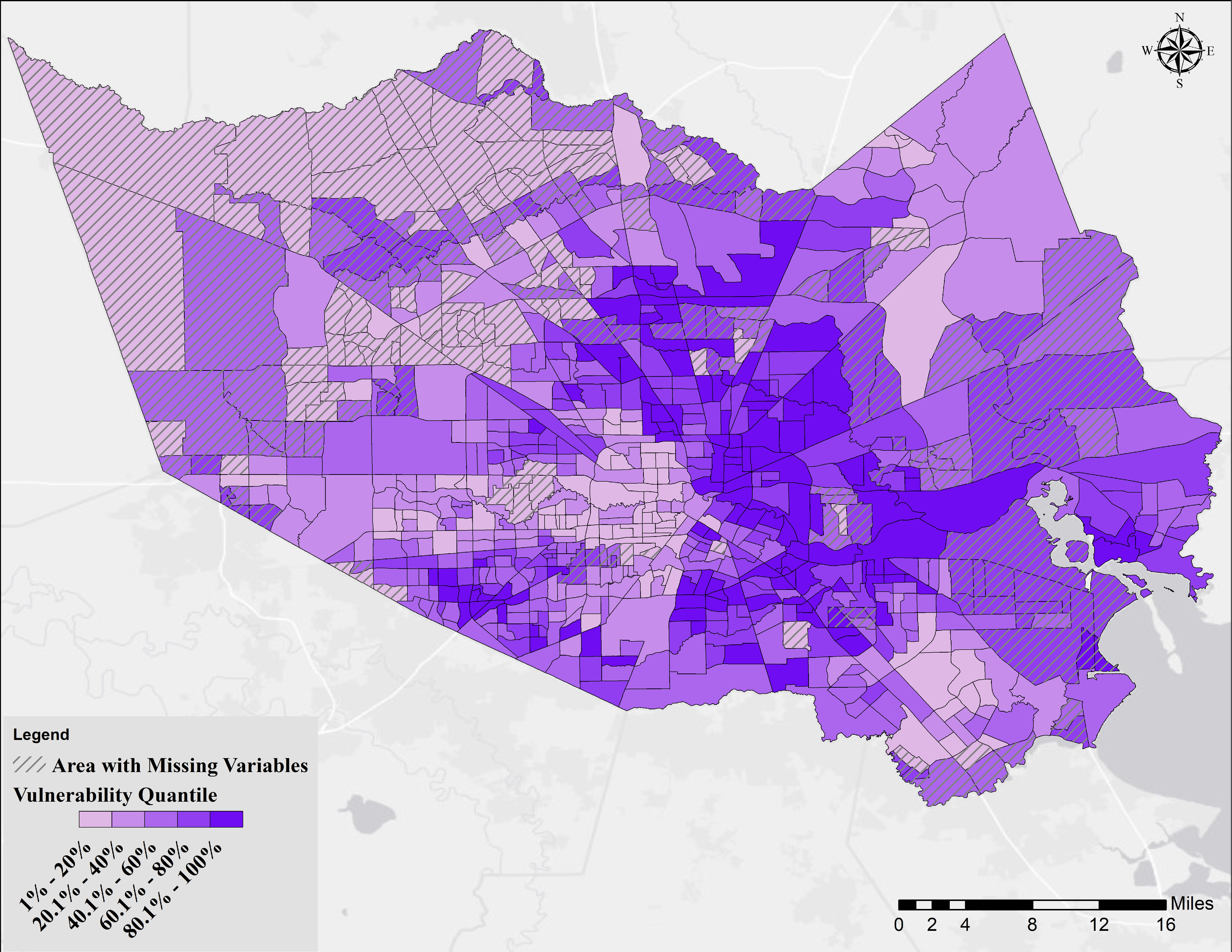
**

**S13 Fig. Results of the K-means analysis method. The graduated colors reflect the percentile of the overall vulnerability. Areas with hatched lines represent census tracts with missing data on chronic disease risk factors, health outcomes, and clinical preventive service form [1]**

**
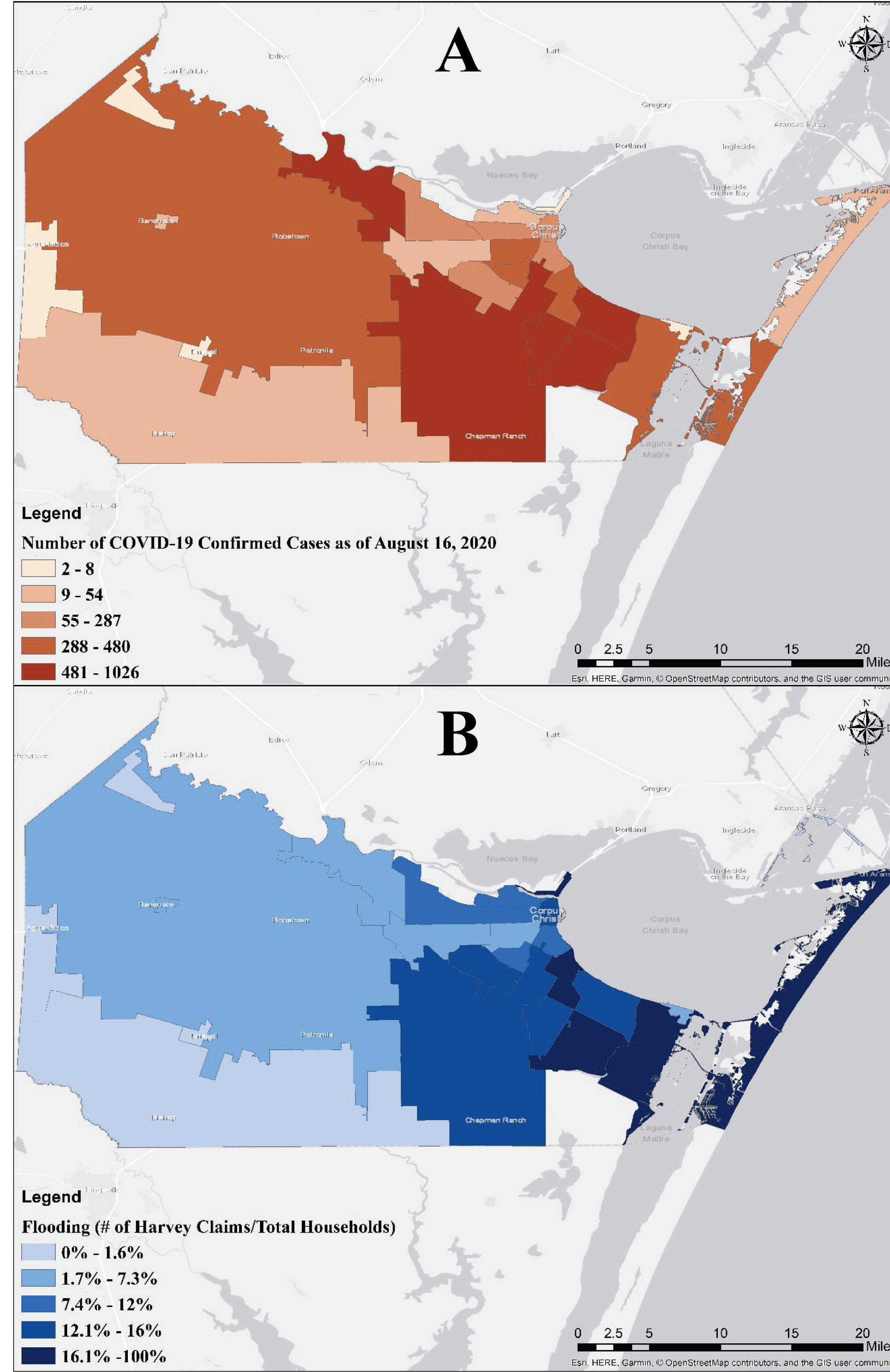
**

**S14 Fig. Geospatial correlation between COVID-19 cases and flooding in Nueces County, Texas**

**
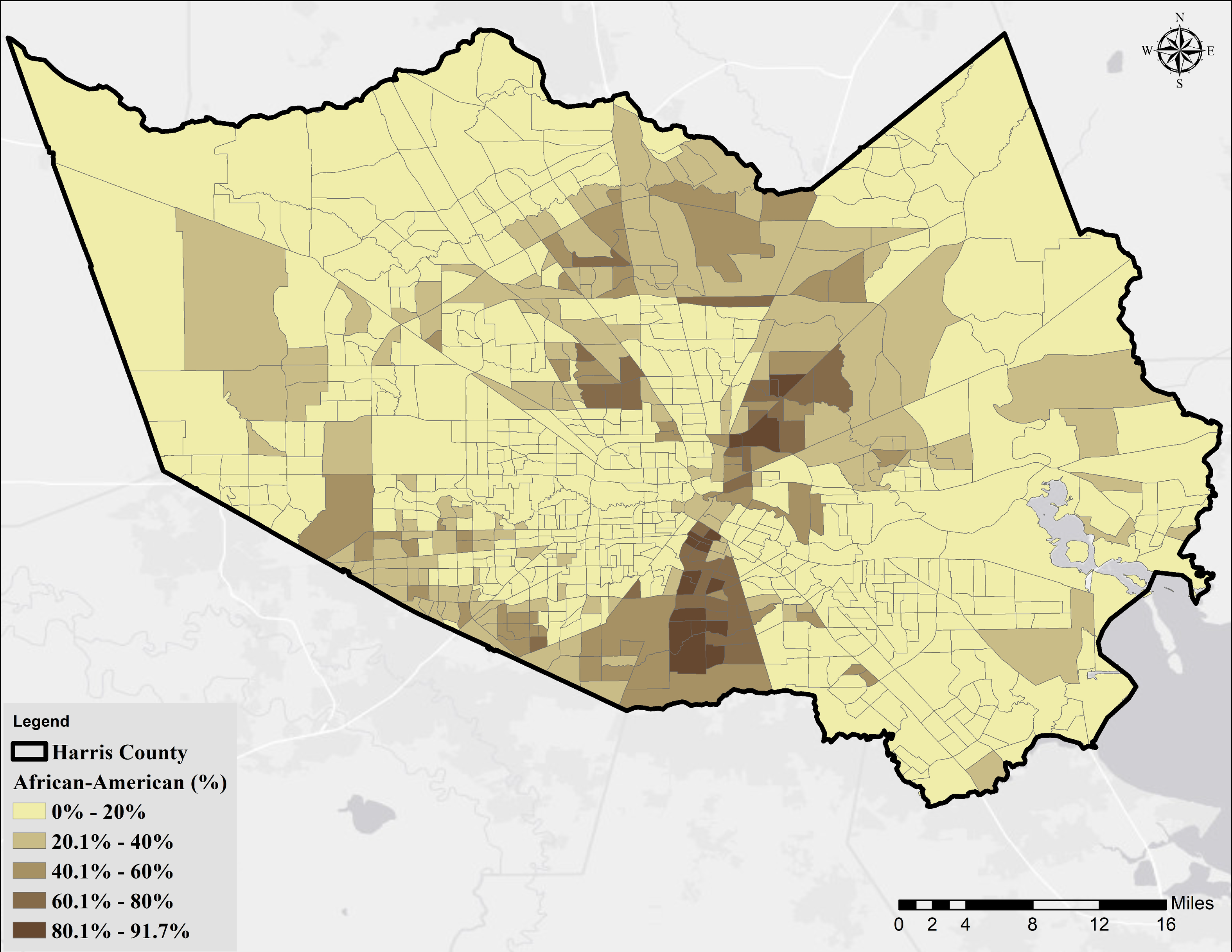
**

**S15 Fig. Geospatial distribution of African-American population in Harris County at the census tract level**

**
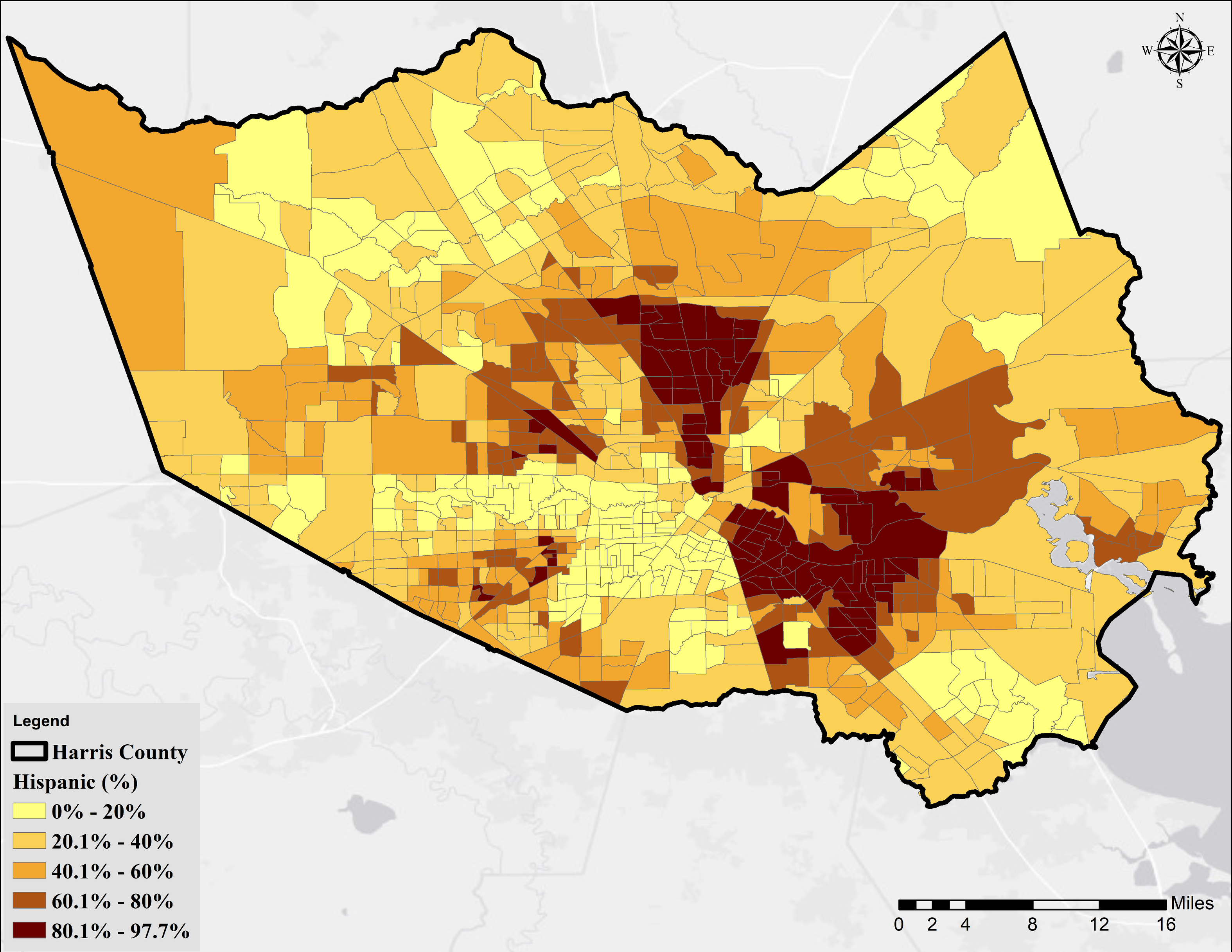
**

**S16 Fig. Geospatial distribution of Hispanic population in Harris County at the census tract level**

**References**

1. HealthLanscape. The 500 Cities Mapper. 2020. Available: https://www.healthlandscape.org/500Cities/
